# Collective Signal Processing in Cluster Chemotaxis: Roles of Adaptation, Amplification, and Co-attraction in Collective Guidance

**DOI:** 10.1371/journal.pcbi.1005008

**Published:** 2016-07-01

**Authors:** Brian A. Camley, Juliane Zimmermann, Herbert Levine, Wouter-Jan Rappel

**Affiliations:** 1 Department of Physics, University of California, San Diego, La Jolla, California, United States of America; 2 Center for Theoretical Biological Physics, Rice University, Houston, Texas, United States of America; 3 Department of Bioengineering, Rice University, Houston, Texas, United States of America; University of British Columbia, CANADA

## Abstract

Single eukaryotic cells commonly sense and follow chemical gradients, performing chemotaxis. Recent experiments and theories, however, show that even when single cells do not chemotax, clusters of cells may, if their interactions are regulated by the chemoattractant. We study this general mechanism of “collective guidance” computationally with models that integrate stochastic dynamics for individual cells with biochemical reactions within the cells, and diffusion of chemical signals between the cells. We show that if clusters of cells use the well-known local excitation, global inhibition (LEGI) mechanism to sense chemoattractant gradients, the speed of the cell cluster becomes non-monotonic in the cluster’s size—clusters either larger or smaller than an optimal size will have lower speed. We argue that the cell cluster speed is a crucial readout of how the cluster processes chemotactic signals; both amplification and adaptation will alter the behavior of cluster speed as a function of size. We also show that, contrary to the assumptions of earlier theories, collective guidance does not require persistent cell-cell contacts and strong short range adhesion. If cell-cell adhesion is absent, and the cluster cohesion is instead provided by a co-attraction mechanism, e.g. chemotaxis toward a secreted molecule, collective guidance may still function. However, new behaviors, such as cluster rotation, may also appear in this case. Co-attraction and adaptation allow for collective guidance that is robust to varying chemoattractant concentrations while not requiring strong cell-cell adhesion.

## Author Summary

To get from one part of the body to another, single cells often follow chemical signals. Sometimes, though, isolated cells ignore these signals, but a group of cells still manages to travel in a directed way. How can this happen? We argue that if the signal changes how the cells interact with their neighbors, a cluster of cells can detect signals single cells ignore. We use computational models to study how this can happen, and show that the speed of the cluster will depend on how the cells process the signal, as well as whether or not the cells are tightly connected to one another. We also show if the cells are only loosely connected, and are attracted to a secreted molecule, cell clusters may develop rotation and other effects that will change how effectively they can sense signals.

## Introduction

Many individual cells, including white blood cells and bacteria, chemotax—sensing and following gradients of signals. Some cells, though, are not loners—they migrate collectively—and cells traveling in clusters and sheets during development must chemotax together. Many experiments [1–5] have shown that clusters can have capabilities that single cells lack: in particular, clusters of cells can follow a gradient even when single cells do not. How can cells work together to follow a gradient that each individual cell is incapable of sensing? How can cells integrate data from across the cluster to improve their gradient sensing abilities? Is cluster chemotaxis essentially different than single-cell chemotaxis? The simplest possibility, that cells just spatially average the gradient signal acting independently on each of them and thereby achieve a more accurate sensing capability, is ruled out, at least for lymphocytes, by experiments that show clusters can travel in the direction opposite to that of single cells [1]. A different possible explanation relies on the qualitative idea of collective guidance [6], in which a cluster of cells can gain a direction even though each of its individual cells senses only the level of signal, and not its gradient. To make this notion more quantitative, we have recently introduced such a model of collective guidance in the context of neural crest cells where the cluster’s directionality comes from a regulation of contact inhibition of locomotion (CIL) [7]; a related model was also proposed for clusters of lymphocytes [1] and extended for studying border cell migration [8].

However, our current understanding of collective guidance and how collective chemotaxis occurs without single-cell gradient sensing does not account for the possibility of response coordinated by chemical signaling between cells. Our minimal model of collective guidance posits that each cell reacts only to the local chemoattractant and the physical presence of its neighbors [7]. More complicated signal processing could take place on the cluster scale if cells use signaling molecules to communicate with each other to collectively process the information contained in the chemoattractant gradient, as was recently suggested to be the case in branching morphogenesis [4, 9]. It is therefore important to ask: What experimental signatures would tell us if this were happening, and how would this signal processing change the efficiency of the cluster’s movement? Can collective signal processing overcome shallow gradients seen *in vivo* (e.g. [10]), amplifying differences in cluster behavior between the front and the back? In minimal models of collective guidance [1, 7], the cluster moves by a tug of war, and is likely under tension. Nevertheless, collective chemotaxis can also occur in the absence of strong adhesion [2]. How does this happen? We will address all of these questions in this paper. Our initial focus will be on understanding *in vitro* experiments in relatively controlled environments [1, 2], especially experiments on explants of neural crest cells, and using these results to develop a useful quantitative framework for the study of collective guidance more generally, including collective chemotaxis *in vivo* [11, 12].

To understand why clusters of explanted neural crest cells chemotax where single cells do not [2], we will analyze both short-range interactions between cells and long-range interactions mediated by chemical secretions. The primary short-range interaction between neural crest cells are cadherin-mediated adhesion and contact inhibition of locomotion (CIL). CIL results in cells repolarizing away from each other after contact. CIL in tissues may be regulated by the type of cadherin expressed, as well as being linked to mechanical force between cells [13–16]. Many possible molecular mediators of CIL have been established, including the non-canonical Wnt-planar cell polarity pathway and ephrin signaling [17, 18]. Within this paper, we will take a phenomenological approach to modeling CIL, describing its consequences rather than its molecular origin.

We first study models of biochemical processing of the chemoattractant signal within the cell cluster, assuming strong cell-cell adhesions as in our earlier model [7]. We treat the possibility of gradient sensing via cell-cell communication, using a mechanism that allows *adaptation*, i.e. the cluster’s response becomes insensitive to the overall level of the signal *S*(**r**). We do this using a local excitation, global inhibition (LEGI) scheme [19]. This model is supported by recent experiments on collective gradient sensing in branching morphogenesis, which identify gap-junction-mediated communication between cells as a critical aspect of collective gradient sensing [4, 9]. We also consider the possibility of cluster-level *amplification* of a sensed gradient, where relatively small changes in the chemoattractant signal *S*(**r**) across the cluster are amplified into much larger changes in the response level. With both adaptation and a switch-like amplification, we find that clusters of an optimal size are more efficient at chemotaxing than either smaller or larger clusters. Amplification of the external signal allows clusters to develop a large velocity even in a shallow gradient. We argue, based on simple scaling principles, that sufficiently large clusters with only short-range adhesion undergoing collective guidance would be expected to either fragment or become increasingly slow.

We then show that if the cohesion of a cluster is not controlled by local cell-cell adhesion, but rather by chemotaxis toward a secreted signal (or “co-attraction” [20]), a cluster of cells can undergo collective guidance by regulation of CIL even if cells are not in continuous contact. We show how co-attraction and regulated CIL interact in order to create robust chemotaxis. In the presence of co-attraction, new behaviors, including persistent cluster rotation, may emerge. We provide an extensive characterization of the transition to rotation, and how rotation can alter the efficiency of gradient-sensing clusters.

## Model

### Collective guidance as driven by contact inhibition of locomotion: quick summary and biophysical motivation

We want to model the collective guidance of a cluster of cells exposed to a chemical gradient *S*(**r**). We use the experiments of [2] on neural crest explants responding to Sdf1 gradients as a guide to determine the features we include as well as the model parameters, though we expect our results to be more generally applicable as well. There are four major elements of a model of this process: 1) single-cell dynamics, 2) physical interactions between cells and contact-range effects like contact inhibition of locomotion, 3) the response of the cells to the chemical *S*(**r**), and 4) chemical communication and signaling between cells.

#### Single-cell dynamics

We model single cell dynamics using a stochastic particle model that, in the absence of other cells, creates an unbiased persistent random walk even in the presence of a chemoattractant gradient. This models the basic observation of [2] that single neural crest cells are relatively insensitive to gradients of Sdf1. Single isolated cells in our model have a behavior that is completely independent of the chemical signal *S*(**r**).

#### Physical interactions and short-range contact inhibition of locomotion

**Fig 1:**
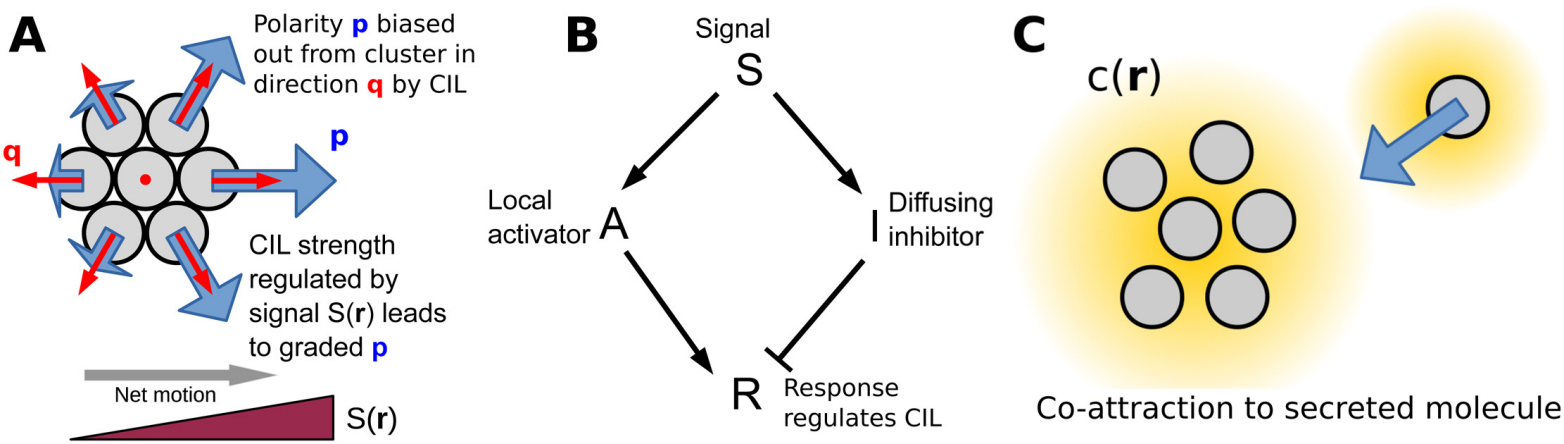
Summary of model mechanisms. (A) Schematic picture of minimal model and origin of directed motion introduced in [7]. Cell polarities are biased away from the cluster toward the direction $q^{i}=\sum_{j∼i}{\hat{r}}^{ij}$ by contact inhibition of locomotion (CIL); the strength of this bias is regulated by the local chemoattractant value *S*(**r**), leading to cells being more polarized at higher *S*. In our minimal model, we assume the bias strength *β* is directly proportional to *S*, but in models with adaptation and amplification, *β* is controlled by the concentration of the response chemical *R*. See text for details. (B) Regulation of CIL strength by signal via a local excitation, global inhibition (LEGI) mechanism. The activator *A* is localized in each cell, while the inhibitor *I* may diffuse between contacting cells. (C) Cluster cohesion may arise from co-attraction, where cells secrete a molecule *c* which diffuses in the extracellular space. Individual cells chemotax up the gradient *c*(**r**).

We include three short-range interactions between cells: cell-cell adhesion, exclusion of overlap between cells, and contact inhibition of locomotion (CIL). CIL is a well-known property of many cell types, notably including neural crest cells, in which cells polarize away from cell-cell contact [15, 21–26]. We model CIL by assuming that the cell polarity, **p**, which describes the cell’s orientation and propulsion strength, is biased away from directions in which the cell touches another cell. The result of this assumption is that, consistent with experiments on neural crest cells [2], the outside edge cells of a cluster are polarized outward, while cells in the interior are unpolarized (Fig 1A).

#### Response of cells to chemoattractant

For a collective guidance mechanism to drive cluster motility, the polarization must be graded across the cluster. As the polarization of the cells at the edge is driven by CIL, we assume that CIL is regulated by the chemoattractant concentration *S*(**r**). This is motivated by the result of Ref. [2], who observe that protrusions on the outside of neural crest clusters are stabilized by the chemoattractant Sdf1. (The assumption that there is an interaction between chemoattractants and CIL is also supported by other recent experiments [18], though we do not explicitly model their results here.) Our earlier minimal model assumed that CIL strength is proportional to the chemoattractant signal *S*(**r**) [7]. We will also model biochemical processing of the signal within cells, including a local excitation, global inhibition (LEGI) mechanism as well as potential amplification of the chemoattractant signal. In the LEGI model, the signal creates both activator and inhibitor molecules within the cell, with the activator localized to each cell and the inhibitor free to diffuse between contacting cells (e.g. via gap junctions). The activator positively regulates the susceptibility of CIL, and the inhibitor negatively regulates it (Fig 1B). All of these mechanisms result in cells at higher values of *S* having a larger susceptibility to CIL, being more polarized, and the cluster moving up the gradient of *S* (Fig 1A).

#### Signaling between cells

We model a potential “co-attraction” between cells as previously seen in neural crest [20, 27]. In this mechanism, single cells both secrete a chemical *c* into the extracellular space and chemotax toward higher levels of *c* (Fig 1C). We note that in our model, isolated cells can chemotax toward the secreted co-attractant *c*(**r**), but do not sense gradients in the signal *S*(**r**). In this way, the concentration *c*(**r**) models the complement fragment C3a, which can lead to single cell chemotaxis [20], while *S*(**r**) represents Sdf1, which does not create single cell chemotaxis [2]. This mechanism can provide cohesion to a cluster even if cell-cell adhesion is completely absent.

### Mathematical description of model

We use a two-dimensional stochastic particle model to describe cells exposed to a chemical gradient *S*(**r**). We describe each cell *i* with a position **r**^*i*^ and a polarity **p**^*i*^. The cell polarity indicates the cell’s direction and propulsion strength, i.e. the velocity with which it would travel in the absence of additional forces; we thus define **p**^*i*^ so that an isolated cell with polarity **p**^*i*^ has velocity **p**^*i*^. The cell’s motion is overdamped, so physical forces like cell-cell adhesion and exclusion change the cell’s velocity—the velocity of the cell is **p**^*i*^ plus the net force the other cells exert on it, $\sum_{j\neq i}F^{ij}$. We model chemically-induced effects like CIL as altering a cell’s biochemical polarity **p**^*i*^. Our model is then:

$$\begin{array}{cc} \partial_{t}r^{i} & =p^{i}+\sum_{j\neq i}F^{ij} \end{array}$$ (1)

$$\begin{array}{cc} \partial_{t}p^{i} & =-\frac{1}{\tau }p^{i}+\sigma \xi^{i}\left(t\right)+\beta^{i}\sum_{j∼i}{\hat{r}}^{ij}\\ & \;\;\;\;\;\;\;\;\;\;\;\;\;+\chi \frac{\nabla c(r^{i})}{\left|\nabla c\right|}\Theta (|\nabla c|-g_{0}) \end{array}$$ (2)

where **F**^*ij*^ are intercellular forces, e.g. cell-cell adhesion and volume exclusion, and ***ξ***^*i*^(*t*) are fluctuating, temporally uncorrelated noise terms that are Gaussian with $\langle \xi_{\mu }^{i}\left(t\right)\xi_{\nu }^{j}\left(t^{\prime }\right)\rangle =2\delta_{\mu \nu }\delta^{ij}\delta \left(t-t^{\prime }\right)$, where the Greek indices *μ*, *ν* run over the dimensions *x*, *y*. The first two terms on the right of Eq 2 are a standard Ornstein-Uhlenbeck model [28, 29]: **p**^*i*^ returns to zero with a timescale *τ*, but is pushed away from zero by the fluctuating noise ***ξ***(*t*). This models a cell that has a motion that is only persistent over a time of *τ*.

#### Cell-cell forces

We adapt the cell-cell force from [30]

$$\begin{array}{c} F^{ij}={\hat{r}}^{ij}\left\{\begin{array}{cc} v_{r}\left(d^{ij}-1\right), & \;\;d^{ij}<1\\ v_{a}\frac{d^{ij}-1}{D_{0}-1}, & \;\;1\leq d^{ij}<D_{0}\\ 0 & \;\;d^{ij}>D_{0} \end{array}\right. \end{array}$$ (3)

where *d*^*ij*^ = |**r**^*i*^ − **r**^*j*^|. This force is a repulsive spring below the equilibrium separation, an attractive spring above it, and vanishes above *D*_0_. We will change *v*_*a*_ and *v*_*r*_ in our simulations to move between clusters that are strongly adherent and those with no short-range adhesion (e.g. *v*_*a*_ = 0).

#### Contact inhibition of locomotion

We introduced the third term on the right of Eq 2 in Ref. [7] to model contact inhibition of locomotion (CIL): the cell’s polarity is biased away from cells near it, toward the vector $q^{i}=\sum_{j∼i}{\hat{r}}^{ij}$, where ${\hat{r}}^{ij}=\left(r^{i}-r^{j}\right)/\left|r^{i}-r^{j}\right|$ is the unit vector pointing from cell *j* to cell *i* and the sum over *j* ∼ *i* indicates the sum over the neighbors of *i* (those cells within a distance of *D*_CIL_ = *D*_0_). For cells along the cluster edge, the direction of the CIL bias (**q**^*i*^) points outward from the cluster, but for interior cells **q**^*i*^ is typically smaller or zero (Fig 1a). Cells around the edge are strongly polarized away from the cluster, while interior cells have weaker protrusions, as observed by [2].

The strength of the CIL bias for cell *i* (i.e. the susceptibility to CIL) is given by *β*^*i*^ in Eq 2. This parameter is regulated by the chemoattractant signal *S*(**r**), as we discuss below.

#### Co-attraction between cells

The final term on the right of Eq 2 is the co-attraction effect: single cells chemotax toward higher levels of *c*, while remaining unable to sense gradients in the signal *S*(**r**). Here, Θ(*x*) is the Heaviside step function, Θ(*x*) = 0 for *x* < 0 and Θ(*x*) = 1 for *x* > 0. This term biases cells to polarize toward increasing *c*, but assumes the strength of this chemotaxis to *c* is independent of the gradient strength, once the gradient strength is above the threshold *g*_0_. The saturation of polarization is supported by recent experiments in T cells [31]. In other cell types other behaviors may be occur [32]; modifying this assumption would change the density and number of contacts in the cluster, leading to potential quantitative changes. We set *g*_0_ = 10^−5^ to be very small; its major role is to prevent division by zero when |∇*c*| is small.

The gradient at the position **r**^*i*^, ∇*c*(**r**^*i*^), is computed under the assumption that secretion, degradation, and diffusion of *c* are much faster than all other processes in our model [27], and is found to be (*Supplementary Information*)

$$\begin{array}{c} \nabla c\left(r^{i}\right)=-\sum_{j\neq i}K_{1}\left(\right|r^{i}-r^{j}\left|/\ell \right){\hat{r}}^{ij} \end{array}$$ (4)

where *K*_1_(*x*) is a modified Bessel function of the second kind and the degradation length *ℓ* is set by *ℓ*^2^ = *D*/*k*_*c*_. We choose *ℓ* to be five cell diameters (100 *μm*, or five in our simulation units), similar to the value estimated and used by [27] in their simulations. We note that the expression above is an approximation that neglects the physical presence of the cells, treating them as pointlike particles; it also treats the space as two-dimensional.

#### Effect and processing of chemoattractant signal

We model the chemical *S*(**r**) as regulating a cell’s susceptibility to CIL, *β*^*i*^. A minimal assumption would be that $\beta^{i}=\bar{\beta }S\left(r^{i}\right)$ [7]. This represents the result of [2] that the cluster chemoattractant Sdf1 stabilizes CIL-induced protrusions [2]. However, we will also allow for the possibility that *β*^*i*^ is regulated in a more complex way:

$$\beta^{i}=\bar{\beta }S\left(r^{i}\right)\;\;\;\left(\text{minimal model}\right)$$ (5)

$$\beta^{i}=\bar{\beta }f\left(R^{i}\right)\;\;\;\left(\text{models with adaptation or amplification}\right)$$ (6)

where here *R*^*i*^ is the concentration of *R* molecules in cell *i*; *R* here is the final read-out of a signal processing network. We will primarily study a simple, adapting model of response to the signal *S*(**r**), the local excitation, global inhibition (LEGI) model (Fig 1B). We generalize LEGI to cell clusters and show that it creates adaptation and gradient sensing. In this LEGI model, signal *S* produces chemicals *A* and *I* within each cell with rates *k*_*A*_, *k*_*I*_. *A* and *I* break down with rates *k*_−*A*_ and *k*_−*I*_. *A* remains localized within each cell, but *I* can be transferred between contacting cells with rate *k*_*D*_. *A* upregulates and *I* downregulates the final output, *R*, which we assume controls CIL, $\beta =\bar{\beta }f\left(R\right)$ (Fig 1B). Our model, which generalizes [19] to clusters, is then:

$$\partial_{t}A^{i}=k_{A}S\left(r^{i}\right)-k_{-A}A^{i}$$ (7)

$$\partial_{t}I^{i}=k_{I}S\left(r^{i}\right)-k_{-I}I^{i}-k_{D}n^{i}I^{i}+k_{D}\sum_{j∼i}I^{j}$$ (8)

$$\partial_{t}R^{i}=k_{R}A^{i}\left(1-R^{i}\right)-k_{-R}I^{i}R^{i}$$ (9)

where *n*^*i*^ is the number of neighbors to the *i*^th^ cell. Eq 8 is a reaction-diffusion model on the network of cells [33, 34]. We note that another group has recently studied a similar LEGI model on cell clusters in the context of branching morphogenesis [4, 9], though in a limited geometry, and without cell-cell rearrangements.

In Eqs 7–9, we have assumed that the inhibitor *I* is transferred diffusively between neighboring cells with a rate *k*_*D*_. Assuming diffusive transfer between contacting cells is appropriate if *I* is transferred from one cell’s cytosol to the other, e.g. by gap junctions. Gap junctions modulate neural crest cell motility *in vivo* [35, 36], making this plausible, though no diffusing inhibitor has yet been identified. If gap junctions do not form quickly enough, it may be possible to create adaptation by extracellular secretions, similar to the processes involved in quorum sensing in bacteria [37] or via “transcytosis” [38]. While we view gap junctions as a likely possibility, we will also occasionally describe our model as “diffusive communication mediated by contact” to not exclude other possibilities.

We also note that we have, in Eqs 7 and 8, assumed that the generation of *A* and *I* is directly proportional to *S*; this assumes that there is no saturation of the chemosensing receptors on the cell.

Amplification of the chemotactic gradient can be modeled by choosing the function *f*(*R*) in Eq 6. Through this paper, we will study a simplified, switchlike form of amplification, so that the response *f*(*R*) is a fixed large value if *R* is above a threshold value, but near-zero if *R* is below that threshold. The form we use is *f*(*R*) = *g*(*R*/*R*_0_), with $g\left(x\right)=\frac{1}{2}[1+\tanh\{(x-1)/\lambda \}]$. *R*_0_ here is the steady-state value of the response *R* in a constant signal.

### Parameter setting

Throughout this paper, we choose our units to be defined by the typical parameters of neural crest cells. With this in mind, we take our length scale to be the typical equilibrium cell-cell separation and our time scale to be the relaxation time—this corresponds to setting the cell diameter to be unity and the relaxation time *τ* = 1. To convert between these simulation units and real units, we use values estimated from the experiments of [2]: typical equilibrium cell-cell separation is 20 *μ*m and the typical time over which a cell reorients is roughly 20 minutes, i.e. *τ* = 20 minutes in real units. Within the simulation units we have chosen, measured neural crest cell velocities are on the order of 1, so we choose *σ* = 1. This choice means that the root mean square speed of an isolated cell is ${\langle |V|}^{2}\rangle^{1/2}=2^{1/2}\sigma \tau^{1/2}\approx 1.4$ microns/minute, in good agreement with, e.g. [2].

When we include adaptation, we assume that the kinetics of Eqs 7 and 9 are fast compared with the dynamics of interest, and set them to their steady states, assuming *k*_−*R*_ ≫ *k*_*R*_ and thus *R*^*i*^ = *A*^*i*, *ss*^/*I*^*i*^(*t*). We set the diffusion rate *k*_*D*_ = 4 in our units, corresponding to a time for equilibration of a few minutes, consistent with experiments using FRAP to see equilibration of fluorescent dyes across gap junctions [39]. However, we note that this rate can depend on the identity of the inhibitor, and may also be regulated [40, 41]. We set the rates of generation and decay of the inhibitor to be *k*_*I*_ = *k*_−*I*_ = 1; this is discussed more in the adaptation section. A complete list of parameters and their justifications is included in the *Supplementary Information*, Table S1.

### Numerical methods

We integrate Eqs 1, 2 and 7–9 explicitly with an Euler-Maruyama integrator [42]. The time step varies: for rigid clusters with high adhesion, we choose Δ*t* = 1 × 10^−4^, and for co-attraction simulations we choose Δ*t* = 1 × 10^−3^. Further details about time step selection as well as source code are available in the Supplementary Information.

## Results

### Review of minimal model of collective guidance in strongly adherent cell clusters

In our earlier paper [7], we studied a minimal version of the model described above, with no co-attraction (*χ* = 0) and no adaptation or amplification, i.e. $\beta^{i}=\bar{\beta }S\left(r^{i}\right)$. We briefly note a few results from that paper here, as in some limits, our more complex model will reduce to this model. Under assumptions of cluster rigidity and slow reorientation, the mean drift of a cluster of cells obeying Eqs 1 and 2 is given by

$$\begin{array}{c} {\langle V\rangle }_{c}\approx \bar{\beta }\tau M\cdot \nabla S \end{array}$$ (10)

with the approximation true for *S*(**r**) ≈ *S*_0_ + **r** · ∇*S*. 〈⋯〉_*c*_ is an average over the fluctuating **p**^*i*^ but with fixed configuration and orientation of cells **r**^*i*^. The matrix M depends only on the configuration of cells; formulas for many cluster shapes and sizes are given in [7]. Mean cluster velocity 〈*V*_*x*_〉 saturates at large number of cells *N*. This arises because we have the difference in signal between the front and the back growing as the cluster radius ($∼\sqrt{N}$), while the perimeter of the cluster also grows as $\sqrt{N}$. The force on the cluster then grows as *N* at large *N*, while the effective friction of the cluster grows independently with the number of cells, as *N*—hence the net velocity should behave as ∼*N*^1/2^ × *N*^1/2^/*N* ∼ 1 at large *N*. (Similar scaling arguments are found for the circular cluster limit in [1].) As we move beyond the minimal model, these scaling assumptions may break down, and therefore larger clusters will not necessarily have saturating velocities.

Ref. [7] also provides analytic results for the chemotactic index CI– a measure of the directionality of the cluster motion. This is commonly defined as the ratio of the distance traveled in the direction of the gradient (the *x* direction) to the total distance traveled. To clarify how we average over many realizations of a path, we define CI = 〈*V*_*x*_〉 / 〈|**V**|〉.

### Adaptation and amplification in strongly adherent cell clusters

#### Motivation for adaptation

In our minimal model of [7], the chemoattractant signal is directly transduced into an increased effect of CIL, neglecting any additional internal processing. However, it is well known that eventual responses of a cell are controlled by networks of interacting proteins and other messengers [43]. These networks can allow cells additional robustness in their responses to a wide variety of signals. One aspect of this robustness, in our model, is the ability to maintain cluster stability independent of the signal strength. In our minimal model [7], a larger signal leads to a larger effect of CIL, and a larger tension on a cluster. We found that, eventually, as adherent cell clusters travel up a chemotactic gradient, they break apart—cluster stability is regulated by the signal level *S*(**r**). Some aggregates, including neural crest cells, do scatter, while others like the border cell cluster [44] remain strongly adherent. For scattering to be regulated *independently* of the response to chemoattractant, the strength of CIL must adapt to changing levels of signal *S*(**r**). Adaptation like this is commonly seen in many cellular and sensory responses, where the response to a signal returns to a baseline level when exposed to a persistently elevated signal [19, 45, 46]. Adaptation can also allow for easier amplification of a shallow signal, making it an important feature in the processing of a chemoattractant signal [19].

How can a cluster of cells adapt its responses while maintaining a graded response across the cluster? One answer comes from gradient sensing in single eukaryotic cells: a local excitation, global inhibition (LEGI) model [19, 47–49]. This model has also been supported in the context of collective gradient sensing by recent experiments and theories of branching morphogenesis [4, 9]. We argue that it is a natural and minimal default model of gradient sensing involving chemical communication. The feed-forward motif of LEGI is also one of only two network topologies that can create adaptation [50]—arguing for its minimality. In this section, we will study the basic behavior of a strongly adherent cell cluster responding to a chemoattractant signal by LEGI-mediated collective guidance. Here, we will neglect co-attraction (*χ* = 0) and set our physical cell-cell interactions to be strong enough that the cluster is highly rigid (*v*_*r*_ = *v*_*a*_ = 500).

#### LEGI on a cell cluster creates perfect adaptation to uniform signals

**Fig 2:**
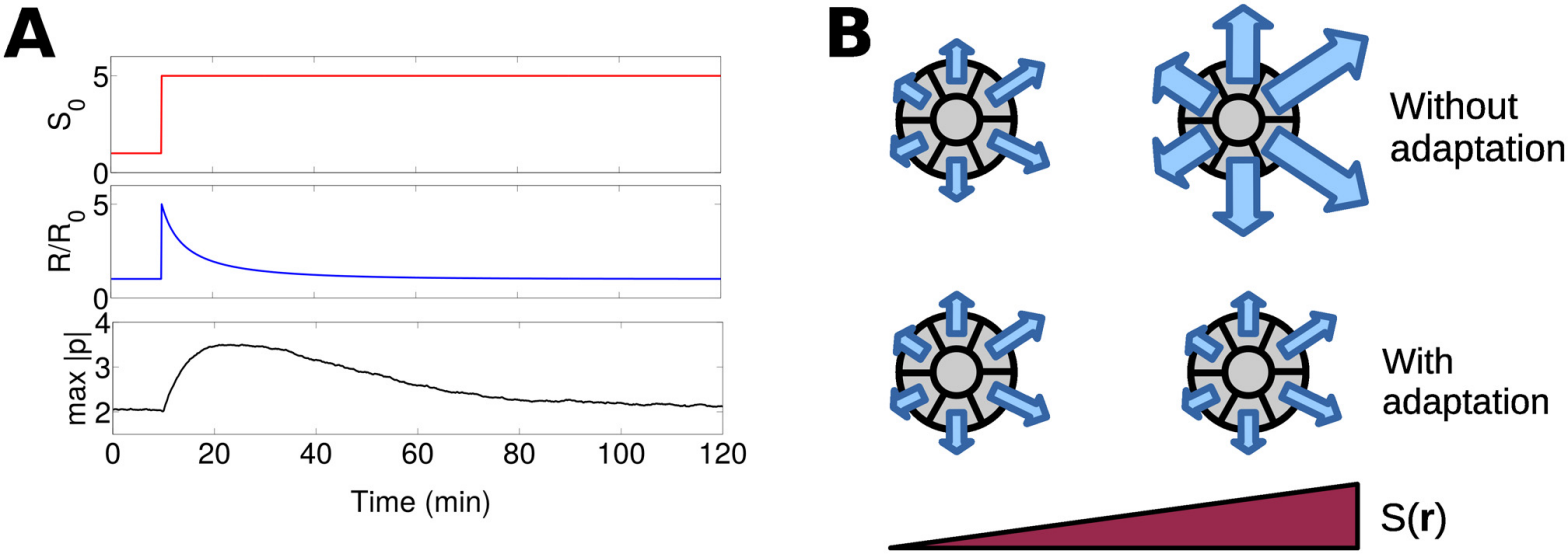
Signal processing via LEGI leads to perfect adaptation and changes cluster stability regulation. (A) Simulation of adaption to an increase in a homogenous signal *S*_0_. Both *R* (which is the same for all cells) and the maximum |**p**^*i*^| respond and then adapt. $R_{0}=\frac{k_{R}}{k_{-R}}\frac{k_{A}}{k_{-A}}\frac{k_{-I}}{k_{I}}$ is the scale of *R* and we assume $\beta^{i}=\bar{\beta }R^{i}/R_{0}$. (B) Schematic of how adaptation allows cluster stability to be regulated independently of cluster chemotaxis. Clusters without adaptation become less stable as they move up the gradient, but those that adapt do not. Arrows are polarity **p**^*i*^.

The LEGI model of Eqs 7–9 perfectly adapts to changing uniform signals, as is shown in the analysis of [19] for LEGI in a single cell. We show this explicitly by finding the steady states of the reaction-diffusion equations. These steady states are simple for *A* and *R*,

$$A^{i,ss}=\frac{k_{A}}{k_{-A}}S^{i}$$ (11)

$$R^{i,ss}=\frac{A^{i}/I^{i}}{A^{i}/I^{i}+k_{-R}/k_{R}}\approx \frac{k_{R}}{k_{-R}}\frac{A^{i}}{I^{i}}$$ (12)

where the approximation holds if the decay rate of *R* is much faster than its creation rate, *k*_−*R*_ ≫ *k*_*R*_. For a signal that is constant in space *S*(*x*) = *S*_0_, $I^{i,ss}=\frac{k_{I}}{k_{-I}}S_{0}$, and thus the steady state *R*^*i*, *ss*^(*t*) is independent of *S*_0_. If *S*_0_ changes over time, *R* first increases and then adapts to its steady-state value, as do the cell polarities (Fig 2A).

#### Ideally adapting clusters develop a velocity that saturates at large cluster size

The LEGI scheme will create a response in the cells that depends on both *S*(**r**) and the chemical kinetics of our reaction-diffusion model. In the limit of fast intercellular diffusion (*k*_*D*_ ≫ *k*_−*I*_) in a connected cluster, $I^{i,ss}\approx \frac{k_{I}}{k_{-I}}\bar{S}$, where $\bar{S}=N^{-1}\sum_{i}S^{i}$ is the mean signal over the cluster (*Supplementary Information*). In this limit and *k*_−*R*_ ≫ *k*_*R*_,

$$\begin{array}{c} R^{i,ss}\approx \frac{k_{R}}{k_{-R}}\frac{k_{A}}{k_{-A}}\frac{k_{-I}}{k_{I}}\frac{S(r^{i})}{\bar{S}}\equiv R_{0}\frac{S(r^{i})}{\bar{S}} \end{array}$$ (13)

Under these assumptions, *R*^*i*^ develops a profile across the cell proportional to the percentage change in the signal *S*(**r**) across the cell. In this limit, if the CIL strength is proportional to *R*^*i*^, $\beta^{i}=\bar{\beta }R^{i}/R_{0}$, we find that $\beta^{i}=\bar{\beta }S\left(r^{i}\right)/\bar{S}$; the strength of polarization is proportional to the local signal divided by the mean signal over the cluster. This is highly similar to our minimal assumptions—the only difference is that the CIL strength *β*^*i*^ is rescaled by the mean signal level over the cluster. Therefore, we would expect Eq 10 and its associated results to apply, but with $\nabla S\to \nabla S/\bar{S}$. In particular, we note that this also predicts that the velocity 〈*V*_*x*_〉 will saturate to a characteristic value at large cluster size, as in the minimal model. However, we caution that in a linear gradient, $\nabla S/\bar{S}$ is not independent of the cluster’s position. For this reason, we generally study adaptation in a shallow exponential gradient, $S\left(x\right)=S_{0}e^{xS_{1}}$ so that $\nabla S/\bar{S}\approx S_{1}$ is constant (as done experimentally in [51]).

We note that the LEGI adaptation scheme functions ideally if intercellular diffusion is fast: a cluster can sense a gradient, as in the minimal model, but the response *β*^*i*^ no longer grows without limit as the cluster travels up the gradient (Fig 2B).

#### Slow diffusive communication can create a non-monotonic dependence of cluster velocity on cluster size

We showed above that in the limit of infinitely fast intercellular diffusion, applying the LEGI adaptation mechanism does nothing more than rescale the response by the mean signal across the cluster. However, in reality, the approximation of infinitely fast diffusion is not realistic. What constraints do the kinetics of diffusive communication place on the LEGI mechanism? We have chosen transfer times to be on the order of a few minutes, corresponding to FRAP experiments on transfer of fluorescent dye through gap junctions [39]; we estimate *k*_*D*_ ≈ 0.2 min^−1^ (*k*_*D*_ = 4 in our units). (We note that [4] estimates a much faster rate, which will depend on the identity of the messenger—their values are estimated for calcium and IP3, while ours is based on [39].) For effective gradient sensing, *I* must equilibrate over the cluster within the timescale 1/*k*_−*I*_, i.e. *α* ≡ *k*_−*I*_/*k*_*D*_ ≪ 1/*N* (*Supplementary Information*).

In principle, *α* could be decreased arbitrarily and we could reach the effectively infinite diffusion limit *α* ≪ 1/*N* just by making the inhibitor degradation rate *k*_−*I*_ increasingly small. However, we would not expect *k*_−*I*_ to be significantly slower than the cell polarity relaxation rate *τ*^−1^. If *k*_−*I*_ ≪ *τ*^−1^, *R* will not reach a steady state over the relevant timescales for cell polarity and motility. We thus expect *k*_−*I*_ ≥ *τ*^−1^. Based on this estimate and the experimental rates for diffusion via gap junctions, we expect *α* ≥ 0.25. This value of *α* is roughly consistent with the experimental findings of [4], though they have estimated significantly faster rates for *k*_*D*_ and *k*_−*I*_. We choose *k*_*I*_ = *k*_−*I*_ = *τ*^−1^ to minimize *α*; we will also show a few examples with larger *α*.

**Fig 3:**
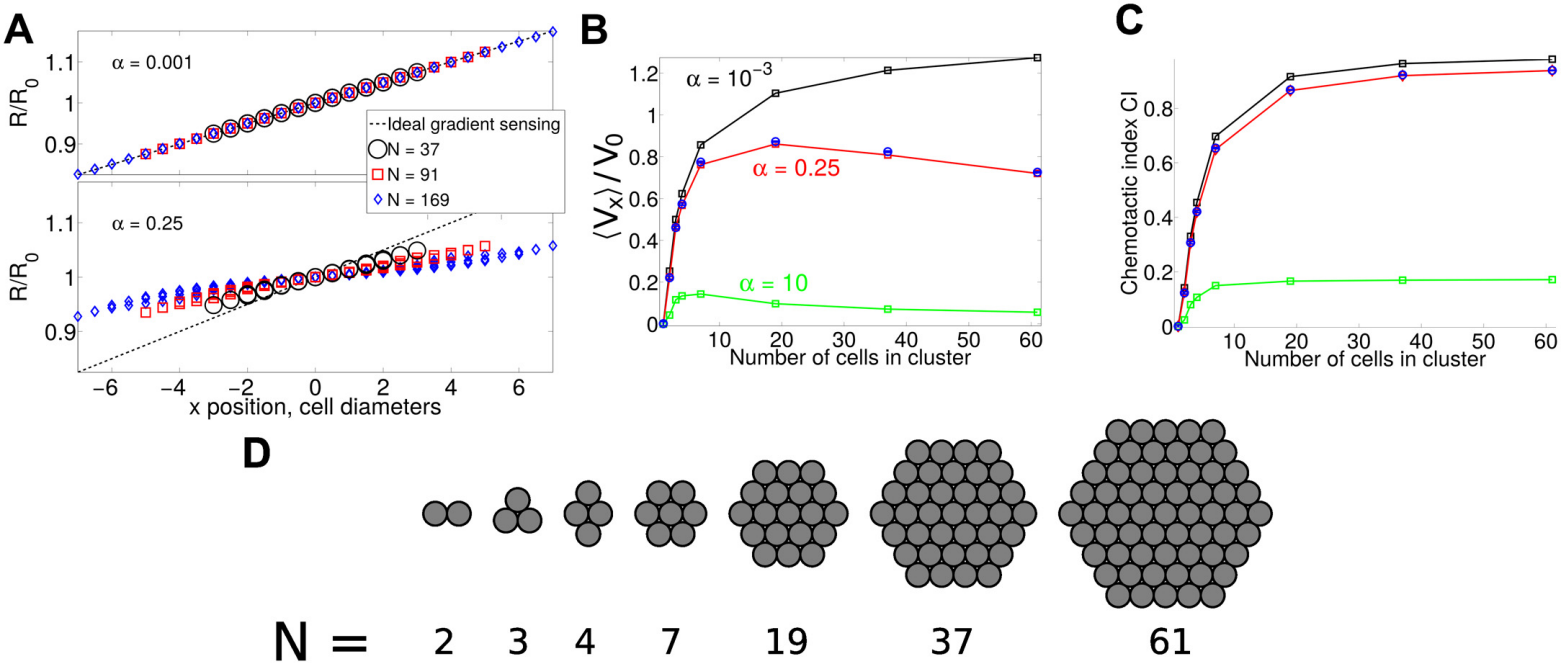
Slow diffusive communication leads to optimum size for clusters using LEGI adaptation. (A) *R*^*ss*^ on clusters of different sizes shown for *α* = *k*_−*I*_/*k*_*D*_ = 0.25 (plausible for gap junction transfer), and *α* = 10^−3^ (near-ideal) in response to a linear gradient. Dashed line shows the ideal result $R^{ss}=R_{0}S\left(x\right)/\bar{S}$. (B) Imperfect LEGI gradient sensing creates an optimal cluster size at which speed is maximized. (C) Chemotactic index of the clusters still increases with increasing *N*. Squares and lines are results assuming rigid clusters and that Eqs 7–9 are at their steady state, while blue circles are full simulations including the reaction dynamics. $V_{0}\equiv \bar{\beta }\tau S_{1}$ is the scale of the velocity. The simulations used to compute (B) and (C) are in an exponential gradient, *S*(*x*) = *S*_0_ *e*^*S*_1_*x*^, *S*_0_ = 1, *S*_1_ = 0.025; *n* ≥ 2000 trajectories of 6*τ* are used for each point. (D) shows the initial cluster shapes assumed in the simulations analyzed in (B) and (C) (though each trajectory starts at a random orientation).

If diffusive communication kinetics are slow with respect to *k*_−*I*_, cells cannot effectively communicate across the cluster, and clusters have imperfect gradient sensing: the gradient of *R* becomes shallower and nonlinear in larger clusters (Fig 3A). When this occurs, cluster velocities change. For *α* = 0.25, mean cluster velocities are non-monotonic in *N*, with a maximum at *N* = 19. This optimum size can be controlled by changing *α* (Fig 3B). Non-monotonicity can make comparison to experiment difficult—using cutoffs for “small” and “large” clusters, as done for some properties in [2], could lead to different results depending on the critical cluster size. Detailed measurements as a function of the cluster radius, as in [1], may be necessary.

We note that the results in Fig 3B and 3C show both full simulations of Eqs 1 and 2 and simplified numerical predictions. We compute the simplified results by assuming, consistent with the minimal analytic model of [7], that the cluster is perfectly rigid, and additionally assuming that reaction-diffusion equations are at their steady state. We can then compute the predicted mean velocity at a particular cluster orientation by ${\langle V\rangle }_{c}=\frac{1}{N}\sum_{i}\langle p^{i}\rangle =\frac{1}{N}\sum_{i}\bar{\beta }f\left(R^{i,ss}\right)q^{i}$; the mean velocity 〈*V*_*x*_〉 is found by averaging over cluster orientation. This also defines the chemotactic index of the cluster.

We show in Fig 3C how the chemotactic index CI changes as a function of the cluster size. CI is a measure of the directionality of the cluster, and is defined by CI = 〈*V*_*x*_〉 / 〈|**V**|〉, where **V** is the cluster velocity (see also discussion in [7]). We would expect, based on our results for rigid clusters, that CI is a monotonic function of $\langle V_{x}\rangle /{\langle \delta V_{x}^{2}\rangle }^{1/2}$, where $\langle \delta V_{x}^{2}\rangle =\langle {\left(V_{x}-\langle V_{x}\rangle \right)}^{2}\rangle =\sigma^{2}\tau /N=\langle {\left(V_{y}-\langle V_{y}\rangle \right)}^{2}\rangle$. Therefore, if the cluster velocity decreases at large *N*, CI may still increase if 〈*V*_*x*_〉 decreases slower than *N*^−1/2^. However, we generally find that CI reaches a roughly constant value with increasing numbers of cells in the cluster, and this value depends on the parameter *α* (Fig 3C). This suggests that merely the imperfect gradient sensing occurring from the finite diffusive communication rate will significantly reduce the cluster’s chemotactic index, as well as preventing it from increasing significantly as the number of the cells increases.

Ellison et. al and Mugler et al. [4, 9] have also recently found that gap junction limited communication can play a role in limiting gradient sensing accuracy in the different context of branching morphogenesis. Our theory shows that in a LEGI model, the collective chemotactic index of the cluster saturates with cluster size (Fig 3C), similar to their result that the signal-to-noise ratio in branching morphogenesis (which does not involve motility) saturates at increasing cluster size.

#### Signal amplification can create non-monotonic dependence of cluster velocity on cluster size

Within chemotaxing single cells, small differences in signal are amplified to large differences in behavior between the cell front and back, allowing efficient migration even in shallow chemotactic gradients [19, 47, 52]. Amplification can also increase cluster motility. Clusters move via a tug-of-war mechanism—cells at the cluster back oppose the net motion of the cluster (Fig 1). If these back cells are suppressed, or the polarization of the cells at the front is amplified, cluster velocity increases.

**Fig 4:**
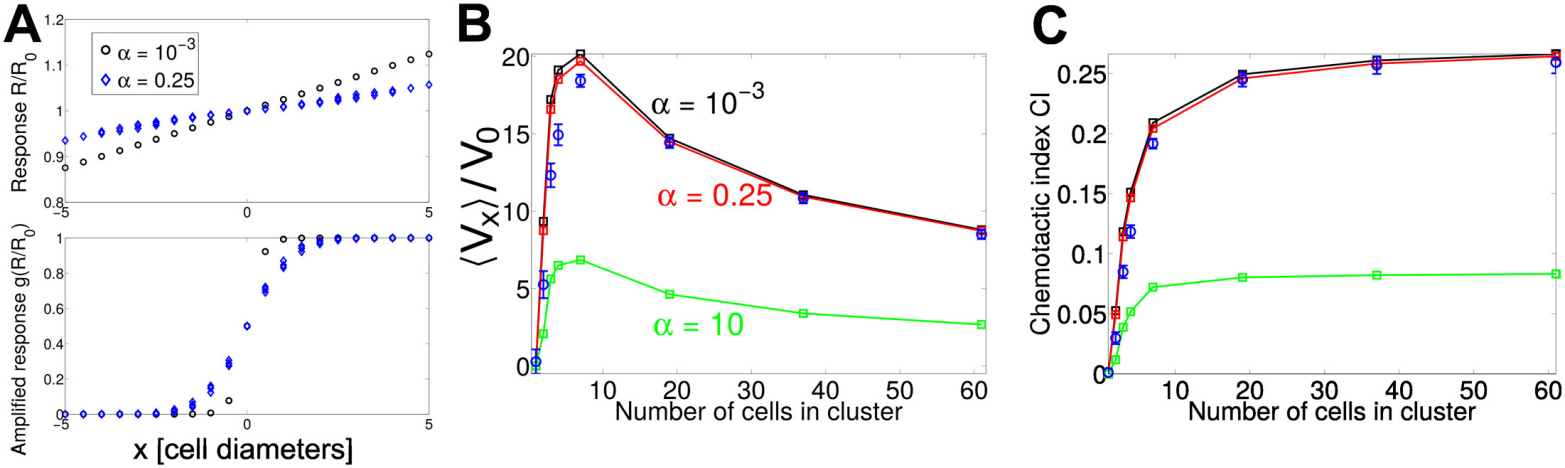
Amplification can reduce effect of diffusive communication kinetics, but also creates an optimal size. (A) *R*^*ss*^ and *g*(*R*^*ss*^) on clusters of different sizes. The use of the switchlike amplification reduces the importance of imperfect gradient sensing, ensuring that the cluster response at the front and the back is the same for either near-ideal (*α* = 10^−3^) and realistic (*α* = 0.25) gradient sensing. (B) Combination of LEGI gradient sensing and amplification significantly increases cluster speed beyond the typical scale $V_{0}\equiv \bar{\beta }\tau S_{1}$ and reduces the dependence of cluster speed on *α*, but creates an optimal cluster size at which speed is maximized. (C) Chemotactic index of the clusters still increases with increasing *N*. Squares and lines are results assuming rigid clusters and that Eqs 7–9 are at their steady state, while blue circles are full simulations including the reaction dynamics. $V_{0}\equiv \bar{\beta }\tau S_{1}$ is the scale of the velocity. The simulations used to compute (B) and (C) are in an exponential gradient, *S*(*x*) = *S*_0_ *e*^*S*_1_*x*^, *S*_0_ = 1, *S*_1_ = 0.025; *n* ≥ 2000 trajectories of 6*τ* are used for each point. $\bar{\beta }=0.2$ in simulations in this figure—larger values of $\bar{\beta }$ can lead to larger deviations from steady-state results. $\beta^{i}=\bar{\beta }g\left(R/R_{0}\right)$, with $g\left(x\right)=\frac{1}{2}[1+\text{tanh}\{(x-1)/\lambda \}]$ and λ = 10^−2^.

We treat an illustrative but extreme example of amplification in which a cell’s response is switchlike, with front cells strongly polarized and back cells suppressed, $\beta^{i}=\bar{\beta }g\left(R/R_{0}\right)$, with $g\left(x\right)=\frac{1}{2}[1+\tanh\{(x-1)/\lambda \}]$. For λ ≪ 1, $\beta^{i}\approx \bar{\beta }$ where *R*^*i*^ > *R*_0_ (cluster front), and *β*^*i*^ ≈ 0 if *R*^*i*^ < *R*_0_ (cluster back). This switchlike response means that the precise value of *R* is not as crucial as whether it is larger or smaller than *R*_0_ (Fig 4A). For this reason, with strong amplification (λ = 10^−2^), cluster velocity is, assuming a steady state of *R*^*i*^, much less sensitive to the intercluster diffusion rate *k*_*D*_ (Fig 4B). However, the assumption that *R*^*i*^ is at its steady state is not necessarily reasonable for amplified clusters; fluctuating *R*^*i*^ coupled with the nonlinear dependence of *β*^*i*^ on *R*^*i*^ above can lead to deviations from the steady-state result (Fig 4B, blue circles). In fact, to see reasonable agreement between the steady-state and full kinetics simulations, we had to reduce $\bar{\beta }$ from its value of 20 in Fig 3 to $\bar{\beta }=0.2$ in Fig 4.

With amplification, cluster velocity increases beyond its usual scale of $V_{0}=\bar{\beta }\tau S_{1}$, as the cluster is no longer engaged in a tug-of-war. However, cluster velocity is still nonmonotonic in cluster size (Fig 4B), decreasing as *N*^−1/2^ at large *N*. This is reasonable, based on a simple scaling argument. As in the minimal model, polarity is only large along the cluster edge (∼*N*^1/2^ cells). However, with amplification, the polarity strength at the edge is *independent of N*—so the mean force driving the cluster scales as *N*^1/2^, while the friction of the whole cluster scales as *N*, leading to 〈*V*_*x*_〉∼*N*^−1/2^. We see again that the chemotactic index does not significantly increase with cluster size as *N* increases from 7 to 61 cells (Fig 4C). This is expected when 〈*V*_*x*_〉∼*N*^−1/2^. Other possibilities for amplification (e.g. $\beta^{i}=\bar{\beta }{\left(R^{i}/R_{0}\right)}^{2}$ [19]) will lead to different behaviors for 〈*V*_*x*_〉 as a function of *N*.

### Clusters bound only by co-attraction

Until this point, we have only looked at highly adherent, effectively rigid clusters. However, collective cell migration can also occur with a high degree of fluidity and cell-cell rearrangement [12, 53–60]. In addition, we have until now assumed that the only attraction between cells is short-range, representing cell-cell adhesion. However, neural crest cells also attract one another through chemical secretions, which can control the extent of cluster directionality and cohesion [20, 27]—and many other cell types also chemotax toward secretions [61, 62]. We extend our model to allow for this possibility, and show that clusters of cells that cohere via co-attraction can also be directed by collective guidance. These clusters need not be rigid, and can have significant re-arrangement or even only transient contacts.

In this section, we will treat clusters with co-attraction (*χ* ≠ 0), but assume only the minimal model of signal processing, with the CIL susceptibility $\beta^{i}=\bar{\beta }S\left(r^{i}\right)$.

#### Clusters with only transient cell-cell contacts can still chemotax effectively

**Fig 5:**
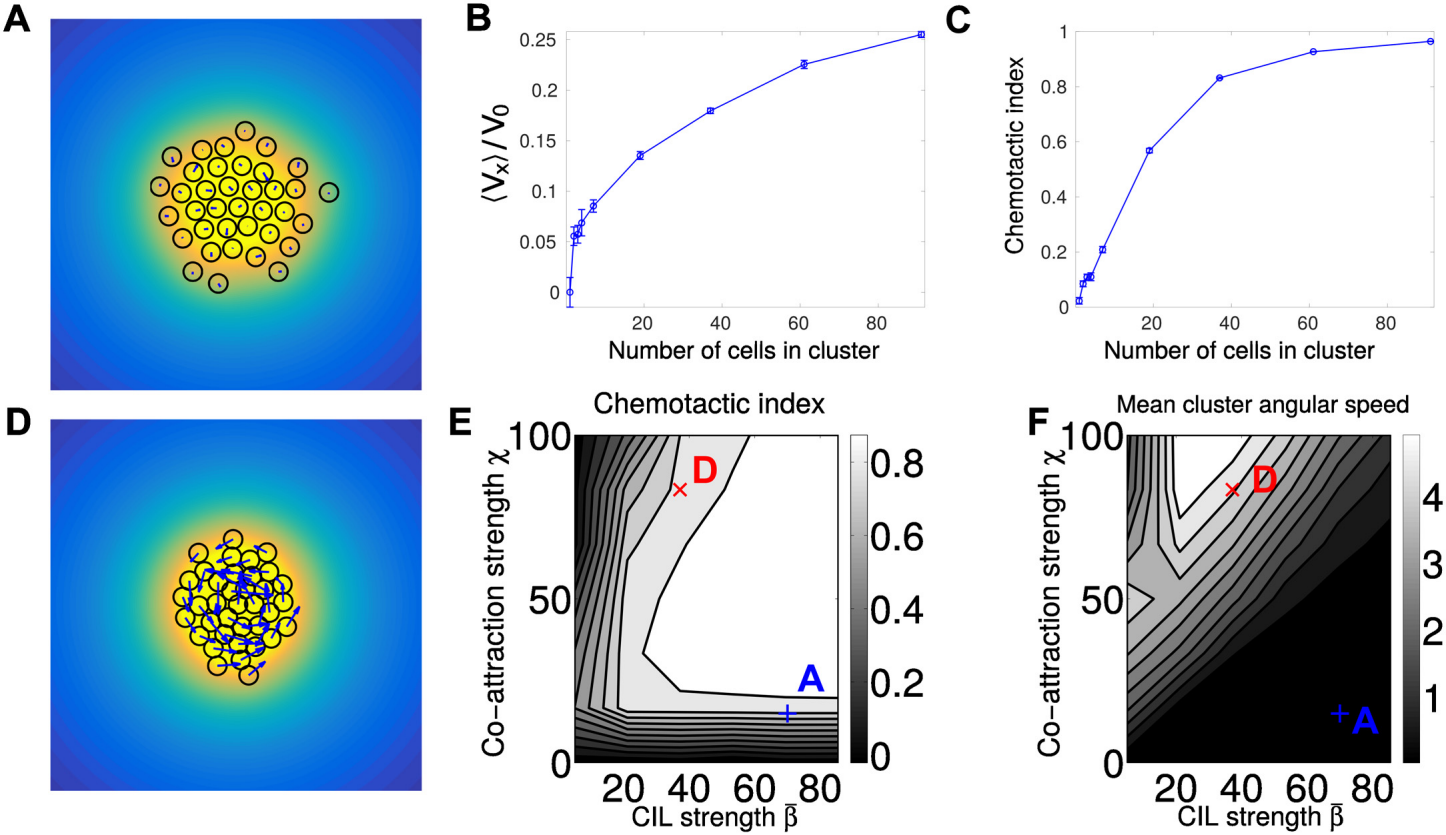
Co-attraction and graded CIL can create directed motion. (A) is a representative snapshot of a chemotaxing cluster loosely bound by co-attraction, with $\bar{\beta }=70$ and *χ* = 15, corresponding to S1 Movie. (B) and (C) show the velocity and chemotactic index of a cluster with these parameters as a function of number of cells in the cluster. Both cluster velocity and CI increase with increasing cluster size; $V_{0}=\bar{\beta }\tau \left|\nabla S\right|$. (D) is a snapshot of a rotating chemotaxing cluster with stronger co-attraction and weaker CIL ($\bar{\beta }=37.2$ and *χ* = 83.3), corresponding to S2 Movie. In (A) and (D), the color map is the co-attractant field *c*(**r**), the blue arrows are the cell polarity **p**^*i*^, and the cells are drawn as black circles. (E) Phase diagram of chemotactic index of clusters of *N* = 37 cells. CI increases when both co-attraction *χ* and CIL strength $\bar{\beta }$ are increased. (F) Phase diagram of mean angular speed 〈|Ω|〉 of clusters of *N* = 37 cells. Clusters with sufficiently high co-attraction develop rotational motion. Points corresponding to the simulations shown in (A) and (D) are marked on the phase diagrams of (E) and (F). Throughout this figure, the degradation length *ℓ* = 5 cell diameters. *n* = 100 trajectories of length 50*τ* are used for each point of the phase diagrams in (E) and (F), which are contour plots based on a 6 × 7 sample of the space $\bar{\beta }\in \left[5,85.6\right],\chi \in \left[0,100\right]$. Varying numbers of trajectories are used for each point in (B) and (C) ranging from *n* = 100 for smaller clusters to *n* = 15 for *N* = 91 Δ*t* = 0.001, *v*_*a*_ = 0, *v*_*r*_ = 100, and |∇*S*| = 0.025 throughout this figure.

Above, we studied one extreme of cell cluster cohesion: highly cohesive clusters linked by short-range adhesion. We now study the opposite limit: no short-range adhesion (*v*_*a*_ = 0), and cluster cohesion solely by chemotaxis to the secreted molecule *c*. Cluster chemotaxis via collective guidance can still function even in this limit (Fig 5A–5C, S1 Movie).

#### Loosely bound cluster chemotactic velocity increases with cluster size

Some qualitative results of our minimal, rigid model are recapitulated in the model with co-attraction, though there are important differences. Larger clusters are generally faster and more efficient (Fig 5B and 5C). Mean velocity increases sublinearly with cluster size. However, unlike [7], we do not observe the speed of the cluster saturating with cluster size. Why? The saturation of the cluster velocity for a rigid cluster in the minimal model [7] (or with ideal adaptation, as above) arises because we balance a force due to CIL that is exerted on the edge of the cluster ($∼\sqrt{N}$ cells), and increases linearly with the radius of the cluster ($∼\sqrt{N}$), hence increasing as *N*, with a drag that comes from a linear combination of all the individual cells (∼*N*). In the mechanism with co-attraction, CIL acts at any cell-cell collision—and these are not limited to the edge of the cluster (Fig 5A and S1 Movie).

We note that the co-attraction simulations in Fig 5 assume the minimal model of $\beta^{i}=\bar{\beta }S\left(r^{i}\right)$, with no adaptation or amplification (we study the interaction of the LEGI adaptation mechanism and co-attraction later). For this reason, as the cell cluster travels up the gradient, the mean value of *β* on the cluster increases, which will change the morphology and dynamics of the cluster. The value of the chemotactic index we present is averaged over the time from 12.5*τ* to 50*τ* after the simulation is initialized; changing this averaging range does not qualitatively change the results in Fig 5.

#### Chemotactic index, cluster rotation depend on balance of co-attraction and CIL

As the degree of co-attraction increases, clusters may also develop a persistent rotational motion while they chemotax (Fig 5D and S2 Movie). This is consistent with other simulations that show that self-propelled particles with long-range interactions from chemotaxis or other sources can develop a vortex state [63–65]. We note, however, that this vortex state arises even though there is no explicit effect that acts to align a cell’s polarity with its neighbors, and can occur even without CIL. We provide a simple explanation for the emergence of cluster rotation in the next section.

We show a phase diagram of the cluster chemotactic index as well as the mean angular speed in the cluster in Fig 5E and 5F. The chemotactic index is generally maximized when the co-attraction strength *χ* and CIL strength $\bar{\beta }$ are similar, and can be increased by simultaneously increasing *χ* and $\bar{\beta }$. We can understand many of these results intuitively. Increasing the co-attraction increases the number and duration of interactions, and since the chemotactic response to the signal *S* emerges from cell-cell interactions, this increases chemotactic efficiency. Increasing $\bar{\beta }$ increases the (graded) polarization of the cells due to CIL, and hence the chemotactic index—but unless *χ* also increases, the increase in CIL causes the cluster density to decrease, reducing the number of interactions. However, increasing *χ* to be much larger than $\bar{\beta }$ leads to both rotation and a decreased chemotactic index. We emphasize that Fig 5C and 5E plot the *cluster* chemotactic index; for loosely bound clusters, especially with rotation, the chemotactic index of an individual cell can be very different from that of the cluster. We also plot the mean angular speed 〈|Ω|〉 of the cluster, computed by first averaging the angular velocity of each cell, taking the absolute value, and then averaging over many iterations.

#### Cluster rotation emerges at large co-attraction via a pitchfork bifurcation

**Fig 6:**
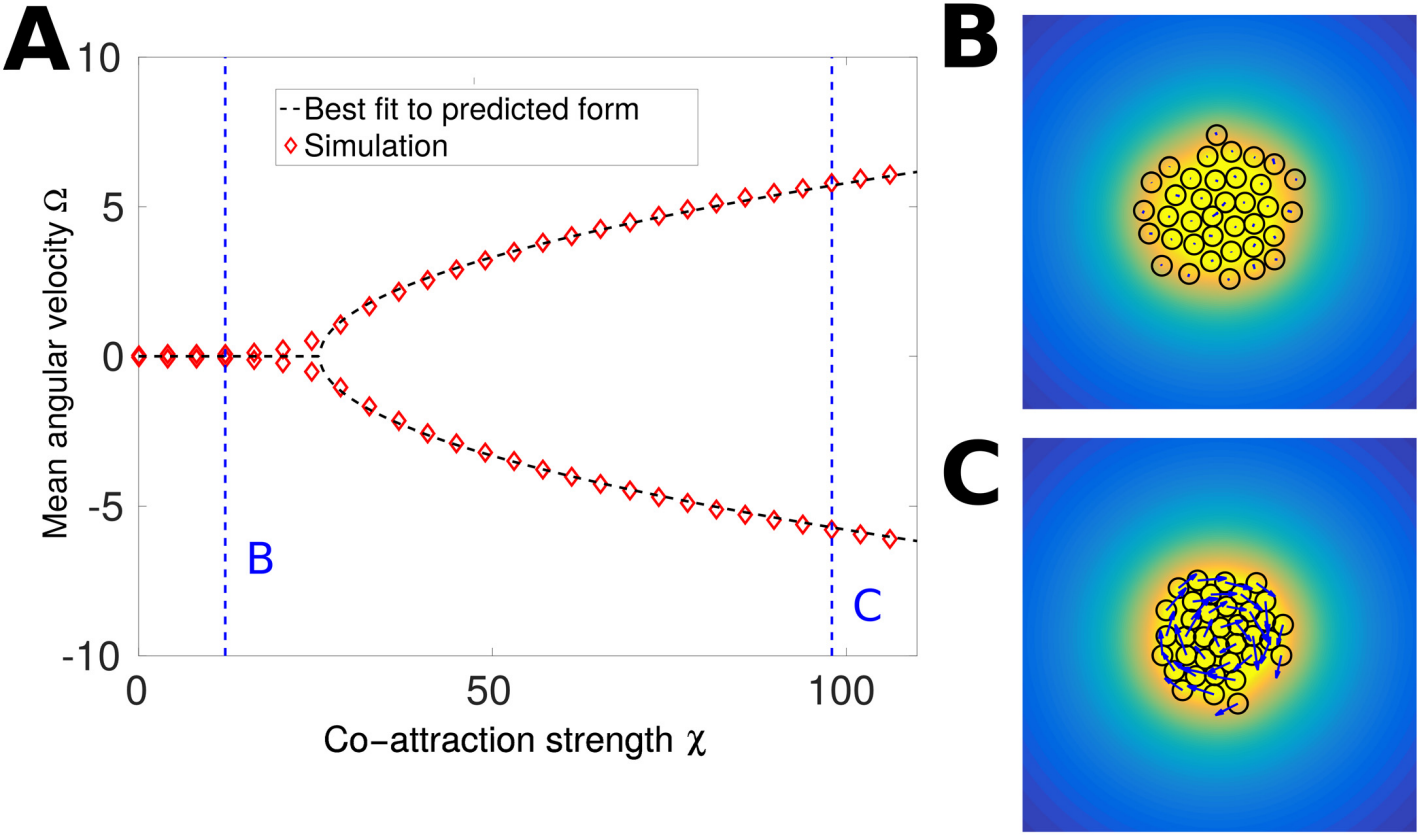
Cluster rotation with co-attraction emerges as a pitchfork bifurcation. (A) The mean angular velocity for our full simulation of clusters under co-attraction is in good agreement with the general form of Eq 21. These trajectories are simulated for *N* = 37 cells with $\bar{\beta }=35$. The two branches in the simulation (A) are the means over those clusters with positive (negative) angular velocity. *n* = 100 simulations of length 50*τ* are used for each value of *χ*. The best fit parameters are Ω_0_ = 0.671 and *χ*_*c*_ = 25.6. |∇*S*| = 0 in these simulations. We highlight two points and show typical simulations in (B) and (C). In (B), *χ* = 12.2, co-attraction is weak, and contacts are only transient. In (C), *χ* = 98.0, and the cluster becomes tightly packed and rotates. In (B) and (C), the color map is the co-attractant field *c*(**r**), the blue arrows are the cell polarity **p**^*i*^, and the cells are drawn as black circles. The vector field **p**^*i*^ has a consistent scale in (B) and (C)—the magnitude of **p**^*i*^ is larger in (C) due to the higher value of the co-attraction strength *χ*.

We can provide a simple argument for how co-attraction can lead to cluster rotation by using a simplified model that neglects some details of the full interactions; these results are surprisingly effective in characterizing the full model (Fig 6). In the rotational phase, we observe that co-attraction is strong enough to keep the clusters tightly packed (Fig 5D and S2 Movie) and the clusters are roughly circular. We also note that the gradient is not required for clusters to develop rotation, and that the rotation will occur even if stochastic forces are negligible. We therefore suggest a simplified model of the rotational motion using two key features: the finite polarity relaxation time *τ* and the tendency of co-attraction to polarize cells toward the center of the cluster. We describe a circular cluster for simplicity, and treat the cluster as perfectly rigid. While the cluster is not completely rigid and cells undergo re-arrangements, we argue that similar mechanisms must be at play in any effectively incompressible cluster. We intend this simple model to be illustrative, but do not expect it to be quantitatively accurate. We also consider the limit of deterministic motion, where the fluctuating noise in our polarity equations vanishes, *σ* → 0. We then write

$$\partial_{t}p^{i}=-\tau^{-1}p^{i}+\Gamma^{i}{\hat{n}}^{i}$$ (14)

$$\partial_{t}\theta =\mu_{\theta }\sum_{i}R^{i}p^{i}\cdot {\hat{t}}^{i}$$ (15)

Here, ${\hat{n}}^{i}$ is the outward normal to the circular cluster at cell *i*, ${\hat{t}}^{i}$ is the tangent to the circle, and Γ^*i*^ indicates the bias arising from a combination of CIL and co-attraction; Γ^*i*^ may be either positive or negative, and may vary from cell to cell. We assume, however, that these forces are completely radial—as would be appropriate for a circular cluster with a radially symmetric co-attractant concentration *c*(*r*). *θ* is the angle of rotation of the cluster, which is driven by the total “torque” exerted on it, $\sum_{i}R^{i}p^{i}\cdot {\hat{t}}^{i}$; *R*^*i*^ is the distance of cell *i* from the cluster center and *μ*_*θ*_ is a mobility. We can write the normal and tangential directions as ${\hat{n}}^{i}=(\cos[\phi_{i}+\theta ],\sin[\phi_{i}+\theta ])$, ${\hat{t}}^{i}=(-\sin[\phi_{i}+\theta ],\cos[\phi_{i}+\theta ])$, leading to $\frac{d}{dt}{\hat{n}}^{i}={\hat{t}}^{i}\dot{\theta }$ and $\frac{d}{dt}{\hat{t}}^{i}=-{\hat{n}}^{i}\dot{\theta }$. Here, *ϕ*_*i*_ is the angular position of the cell when the cluster is at rest at *θ* = 0. Writing $p^{i}={\hat{n}}^{i}p_{n}^{i}+{\hat{t}}^{i}p_{t}^{i}$, we find

$${\dot{p}}_{n}^{i}-\dot{\theta }p_{t}^{i}=-\tau^{-1}p_{n}^{i}+\Gamma^{i}$$ (16)

$${\dot{p}}_{t}^{i}+\dot{\theta }p_{n}^{i}=-\tau^{-1}p_{t}^{i}$$ (17)

$$\dot{\theta }=\mu_{\theta }\sum_{i}R^{i}p_{t}^{i}$$ (18)

We define $\Omega \equiv \dot{\theta }=\mu_{\theta }\sum_{i}R^{i}p_{t}^{i}$ and $W\equiv \mu_{\theta }\sum_{i}R^{i}p_{n}^{i}$. Multiplying Eqs 16 and 17 by *R*^*i*^ and summing over *i*, we find

$$\dot{W}-\Omega^{2}=-\tau^{-1}W+\mu_{\theta }\sum_{i}R^{i}\Gamma^{i}$$ (19)

$$\dot{\Omega }=-\left[\tau^{-1}+W\right]\Omega$$ (20)

We see immediately two steady states. The first steady state has no rotation, Ω = 0, and *W* = *μ*_*θ*_ *τ*∑_*i*_ *R*^*i*^ Γ^*i*^ ≡ *W*_0_. The second steady state has constant rotational motion, with Ω = ±[−*τ*^−2^−*μ*_*θ*_∑_*i*_ *R*^*i*^ Γ^*i*^]^1/2^ = *τ*^−1^[−1−*W*_0_ *τ*]^1/2^ and *W* = −*τ*^−1^. For *W*_0_ *τ* < −1 (i.e. Γ^*i*^ sufficiently negative) Ω is real. Linearizing around the no-rotation steady state, *W* = *W*_0_+*δW*, Ω = 0+*δ*Ω, we find that the non-rotating state also becomes unstable for *W*_0_ *τ* < −1. We thus expect that we see rotation once Γ becomes sufficiently negative—i.e. the co-attraction becomes strong compared with the CIL. In our larger model, the effect biasing our polarities toward the center of the cluster arises only from co-attraction—so we expect that in our full model, the role of Γ will be played by −*χ*. In this case, we would expect

$$\begin{array}{c} \Omega =\left\{\begin{array}{cc} 0 & \chi \leq \chi_{c}\\ \pm \Omega_{0}\sqrt{\chi -\chi_{c}} & \chi >\chi_{c} \end{array}\right. \end{array}$$ (21)

where Ω_0_ and *χ*_*c*_ will depend on the details of the model, e.g. the extent of CIL and the number of cells. We show in Fig 6 that Eq 21 is a very good description of how our full model with co-attraction transitions to rotation. Clusters transition to rotation at a critical *χ*_*c*_, which for the parameters of Fig 6 is found to be *χ*_*c*_ ≈ 25.6, and the angular velocity of clusters increases as $\sqrt{\chi -\chi_{c}}$ as *χ* increases. We have seen deviations from Eq 21 at larger *χ*, in which the cluster breaks apart. However, we also found that in this region of parameter space (*χ* > 120), our results have not been numerically robust, so we do not present them here.

We also emphasize that this effect occurs in part because we are not resolving torques on individual cells. As the cluster rotates, the cell’s polarity **p**^*i*^ is constant in the frame of the substrate, rather than the frame of the rotating cluster, i.e. individual cells are free to rotate in place. The collective rotation may therefore be modified or absent in models that resolve, e.g. torques on individual cells or full cell structure; this is an area of future interest.

Why does the cluster rotation only occur when the cluster is held together by the co-attraction, and not by mechanical forces like the adhesion? The critical difference between these two cases is that within our model, mechanical forces act on the cell’s velocity (Eq 1)– but do not influence its biochemical polarity **p**^*i*^. By contrast, the co-attraction leads the cluster to develop an inward polarization.

#### Rotating clusters develop chirality-dependent drift

**Fig 7:**
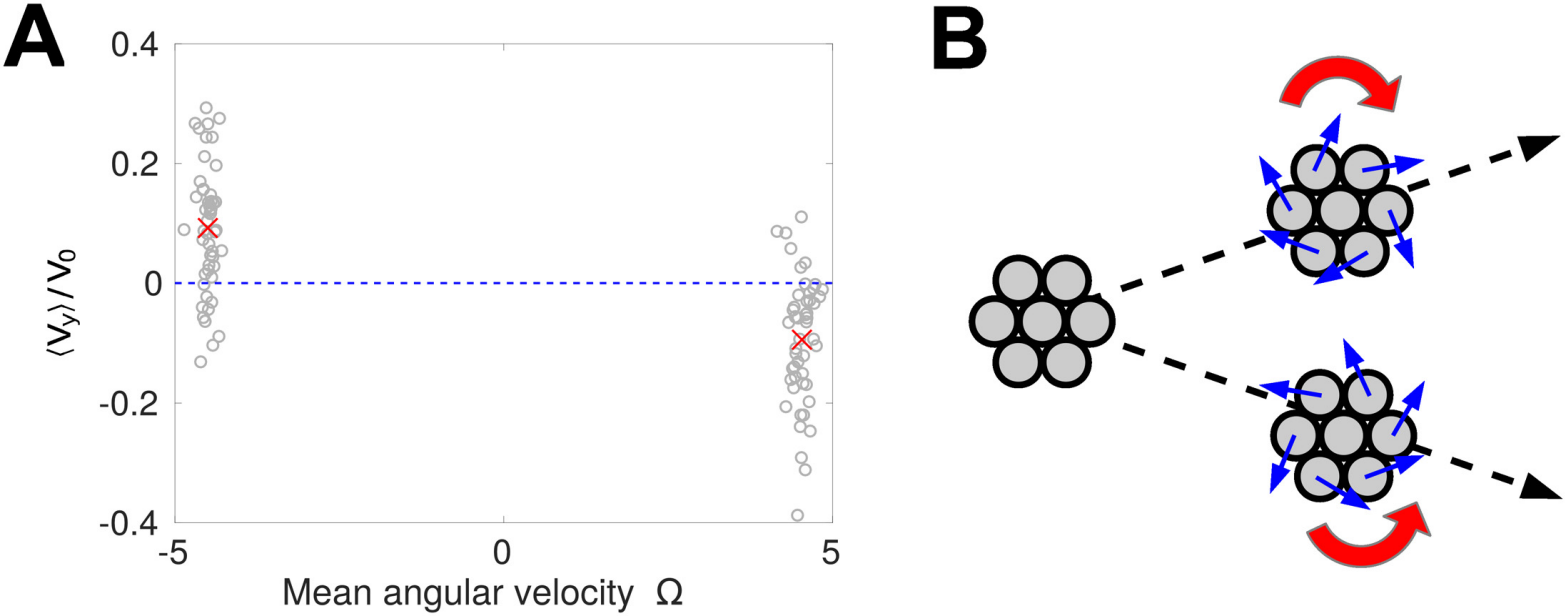
Rotating clusters develop a handedness-dependent drift perpendicular to the gradient. (A) The mean drift velocity 〈*V*_*y*_〉 for 100 trajectories is plotted as a function of that trajectory’s mean angular velocity (gray circles). The mean drift velocities for the clusters with positive and negative angular velocities are plotted as red crosses. These trajectories are simulated for *N* = 37 cells with the parameters of Fig 5D, with the averages evaluated over the time from 12.5*τ* to 50*τ* after the simulation is initialized, as in Fig 5D. (B) Schematic picture of cluster trajectories showing that even if the motion is deterministic, the rotation-drift coupling can change the cluster chemotactic index.

When the cluster develops a spontaneous rotation, this changes the underlying symmetries of our problem. Ordinarily, for a roughly circular cluster, our system is symmetric with respect to inversions in the *y* direction (the direction perpendicular to the chemoattractant gradient). This suggests that the net drift in the *y* direction should be zero. However, the rotation of the cluster gives it a handedness that breaks the *y* inversion symmetry—allowing a net drift in the *y* direction, whose sign would then depend on the handedness of the rotation. We show in Fig 7A that this is the case. This gives a possible explanation for why, in Fig 5E and 5F, the chemotactic index of clusters decreases as cluster rotation begins. As shown schematically in Fig 7B, even if the cluster’s motion is completely deterministic, a systematic drift velocity in the *y* direction will reduce the cluster’s chemotactic index, because the mean motion no longer is purely in the *x* direction.

What is the origin of this drift? We cannot explain this result in terms of the assumptions that led to Eq 21—if we extend this model to have a position-dependent Γ, it will predict that the drift in the y direction is zero. We suspect that this drift in our full model largely arises from co-attraction. Small distortions in the cluster arising from the rotation can lead to a bias for cells that would otherwise see relatively weak gradients. However, we note that this particular mechanism requires cells that are sensitive to weak gradients in the co-attractant, and then amplify this into larger motion. We have found that handedness-dependent drift in our model is sensitive to details such as our assumption that cells respond equally strongly to weak and strong gradients of the co-attractant *c*(**r**). We nevertheless want to highlight the chirality-dependent drift as a feature that is not excluded on symmetry grounds and may arise more robustly in other models.

#### Combination of co-attraction and adaptation

Here, we study the combination of our two models, including both the LEGI adaptation mechanism and co-attraction. Though we treat this question for completeness, we note that our model of short-range contact-mediated diffusive communication is less appropriate for cells without strong short-range adhesion, which do not necessarily have strong gap junctions [66]—though we note for neural crest cells *in vivo* gap junctions are relevant [67]. If gap junctions are not robustly present, the origins of cell-to-cell signal transfer may be different, e.g. transfer via extracellular vesicles or nanotubes [68, 69].

**Fig 8:**
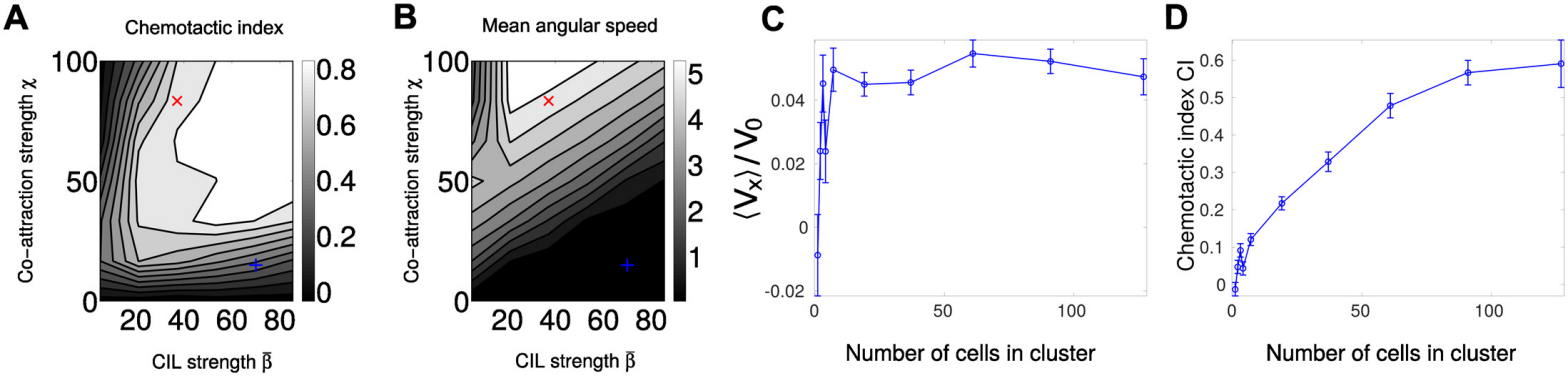
Combination of co-attraction and adaptation does not qualitatively change phase diagram. (A) Phase diagram of chemotactic index of clusters of *N* = 37 cells. CI increases when both co-attraction *χ* and CIL strength $\bar{\beta }$ are increased, as in Fig 5E. (B) Phase diagram of mean angular speed 〈|Ω|〉 of clusters of *N* = 37 cells. Clusters with sufficiently high co-attraction develop rotational motion, as in Fig 5F. (C) Velocity in gradient direction increases, with a dip, as a function of cluster size, but is significantly smaller than the nominal scale $V_{0}\equiv \bar{\beta }\tau S_{1}$. (D) Chemotactic index increases with increasing cluster size. Throughout this figure, the degradation length *ℓ* = 5 cell diameters. *n* = 100 trajectories of length 50*τ* are used for each point of the phase diagrams in (A) and (B), which are contour plots based on a 6 × 7 sample of the space $\bar{\beta }\in \left[5,85.6\right],\chi \in \left[0,100\right]$. Δ*t* = 0.001, *v*_*a*_ = 0, *v*_*r*_ = 100. The gradient is exponential, *S*(*x*) = *S*_0_ *e*^*S*_1_*x*^, *S*_0_ = 1, *S*_1_ = 0.025. (C) and (D) are evaluated at $\bar{\beta }=70$, *χ* = 15 and use varying numbers of trajectories ranging from *n* = 100 for smaller clusters to *n* = 5 for *N* = 127.

We show the phase diagram for how the chemotactic index CI and the mean angular speed depend on the strength of CIL $\bar{\beta }$ and the strength of co-attraction *χ* in Fig 8A and 8B. As in our model with co-attraction, but no adaptation, we see rotation at large *χ* and a chemotactic index that is maximized at large *χ* and *β* (though reduced when rotation occurs).

When we plot the cluster velocity (Fig 8C), we note two features. First, we see that there is an apparent dip in velocity for intermediate numbers of cells, but then a saturation at large cell numbers. This is perhaps not surprising given the combination of adaptation, for which we see a decrease in cluster velocity at large *N* (Fig 3B), and co-attraction, where in the absence of adaptation, the cluster velocity increases consistently with increasing *N* (Fig 5B). Altering adaptation parameters may also lead to different effects of this combination. Secondly, the cluster speed is significantly smaller than seen in Fig 5B, even though the co-attraction and CIL parameters have remained the same. This decrease in speed is a natural consequence of the highly transient contact between cells, as the LEGI mechanism takes time to create a gradient in *R*^*i*^ across an area of cells in contact. In addition, if fewer junctions form between cells with transient interactions (e.g. gap junctions are downregulated), this effect could be aggravated. However, we do see that as the number of cells increases, the cluster is still efficiently directed and CI increases (Fig 8D)

#### Controlling response to changing signal levels by adaptation and co-attraction

**Fig 9:**
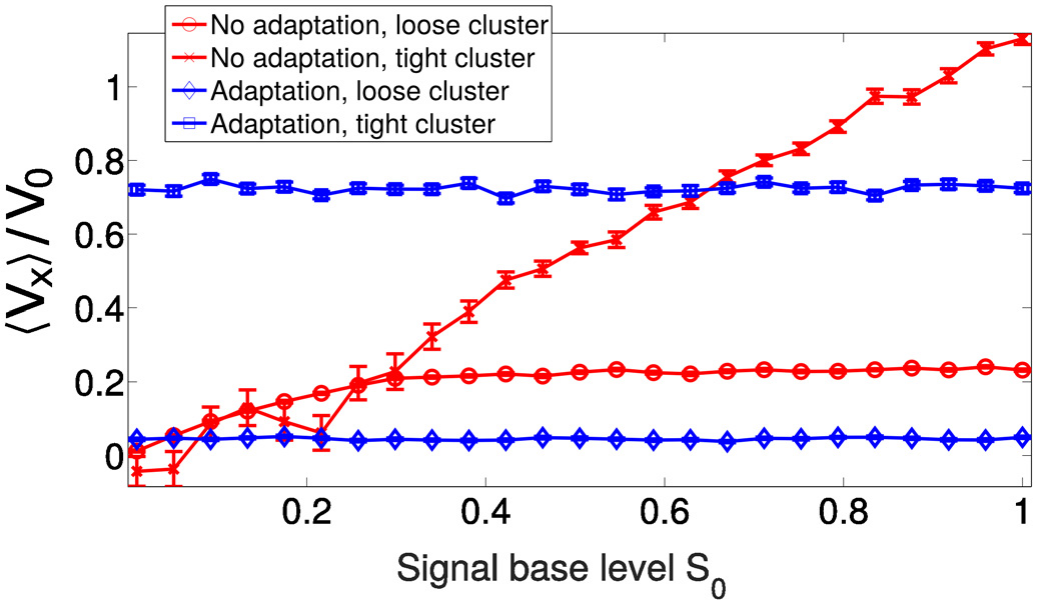
Changing overall signal levels influences cluster speed in the absence of adaptation. $V_{0}\equiv \bar{\beta }\tau S_{1}$. Throughout this figure, the degradation length ℓ = 5 cell diameters. *n* = 100 trajectories of length 50*τ* are used for each value of *S*_0_. Δ*t* = 0.001, *v*_*a*_ = 0, *v*_*r*_ = 100. The gradient is exponential, *S*(*x*) = *S*_0_ *e*^*S*_1_*x*^, *S*_1_ = 0.02. Tight clusters have $\bar{\beta }=37.2$ and *χ* = 83.3; loose clusters have $\bar{\beta }=70$ and *χ* = 15.

While the phase diagrams in Fig 8A and 8B are qualitatively similar between the case with adaptation and without, adaptation can still have a key effect in controlling the response to varying levels of signal. We show the mean velocity of a co-attracting cluster in an exponential gradient *S*_0_ *e*^*S*_1_*x*^ with varying levels of *S*_0_ in Fig 9. When adaptation is present, cluster velocity is independent of this level—as we would expect from the analysis above (e.g. Eq 13 and Fig 2).

In the absence of adaptation, we find that as *S*_0_ is increased, the mean cluster velocity changes (Fig 9). In tightly packed clusters ($\bar{\beta }=37.2,\chi =83.3$, leading to rotating clusters), we see a linear increase in mean cluster velocity up the gradient. This is consistent with our expectations from analytical results on rigid clusters without adaptation or co-attraction [7], which have a velocity proportional to ∇*S*—and hence in a gradient *S*_0_ *e*^*S*_1_*x*^ would have a speed proportional to *S*_0_. For less tightly-packed clusters ($\bar{\beta }=70,\chi =15$), we also see an increase in 〈*V*_*x*_〉 with *S*_0_ at small values of *S*_0_—but then a saturation to a fixed 〈*V*_*x*_〉 at high *S*_0_. In the minimal model without adaptation, $\beta^{i}=\bar{\beta }S\left(r^{i}\right)$, so increasing *S*_0_ is equivalent to increasing $\bar{\beta }$. The saturation in Fig 9 is thus consistent with that seen in Fig 5E, where at fixed co-attraction, CI increases and then saturates with increasing $\bar{\beta }$.

Why does cluster velocity become independent of the signal level in Fig 9 for loosely-bound clusters, even in the absence of an explicit adaptation mechanism? We can understand how adaptation occurs in the absence of LEGI by considering the collision of two isolated cells in the mechanism without LEGI (details in *Supplementary Information*). CIL begins once two cells *i* and *j* reach a critical distance *D*_0_. If $\bar{\beta }$ is sufficiently large, the two cells will quickly be repolarized away from one another along the cell-cell contact axis ${\hat{r}}^{ij}$, as an almost-elastic collision. However, because of the graded CIL response, the cell at larger *x* is more strongly repolarized, and the total polarity **p**^*i*^ + **p**^*j*^ of the pair increases. The time it takes the pair of cells to separate, t_*_, only depends on the average signal over the pair: If two pairs of cells have CIL susceptibilities *β*^*i*^ and *β*^*j*^, the time it takes them to separate scales as t_*_ ∼ 1/[*β*^*i*^ + *β*^*j*^]. By contrast, the total change in **p** due to the collision depends on the difference in signal across the pair, (*β*^*i*^ − *β*^*j*^)t_*_. The combination leads to a net change in polarity—and thus velocity—that only depends on the relative change in signal across the pair. Defining $\Delta \equiv p^{i}-p^{j}$ and $\Sigma \equiv p^{i}+p^{j}$, we show in the *Supplementary Information* that for sufficiently large *β*^*i*^,

$$\Delta \left(t_{*}\right)\cdot {\hat{r}}^{ij}\approx -\Delta \left(0\right)\cdot {\hat{r}}^{ij}$$ (22)

$$\Sigma \left(t_{*}\right)\cdot {\hat{r}}^{ij}\approx \Sigma \left(0\right)\cdot {\hat{r}}^{ij}-2\Delta \left(0\right)\cdot {\hat{r}}^{ij}\frac{\beta^{i}-\beta^{j}}{\beta^{i}+\beta^{j}}$$ (23)

$$\begin{array}{cc} & \approx \Sigma \left(0\right)\cdot {\hat{r}}^{ij}-\Delta \left(0\right)\cdot {\hat{r}}^{ij}\bar{\beta }S_{1}D_{0} \end{array}$$ (24)

where the last approximation holds for a shallow exponential gradient *S*(*x*) = *S*_0_ *e*^*S*_1_*x*^ with *S*_1_ *D*_0_ ≪ 1.

Eqs 22–24 show that the scattering of pairs of cells can, even in the absence of a LEGI mechanism, depend only on the relative gradient, and be insensitive to the overall level *S*_0_. This insensitivity to *S*_0_ arises because *S*_0_ increases the strength of graded pair repolarization—but in proportion decreases the amount of time the pair has to repolarize. Our argument provides a plausible intuitive explanation for the emergence of adaptation in Fig 9. Some assumptions—e.g. purely pairwise collisions—might need to be extended to make this a quantitative theory that could predict the exact velocities shown in Fig 9.

## Discussion

In our earlier work [7], we provided a minimal quantitative model that embodied the collective guidance hypothesis [2, 6] and provides a plausible initial model for collective chemotaxis when single cells do not chemotax. However, this model made two fairly strict assumptions: first, that the cluster does not perform any relevant internal processing of the chemoattractant gradient, and second, that the cluster is highly adherent with cohesion from short-range interactions. We and others have used similar models to analyze collective chemotaxis in many biological contexts [1, 7, 8]. In this paper, we have relaxed both of these assumptions, and showed that new qualitative behaviors may arise, emphasizing potentially necessary extensions to these models.

We find, consistent with our earlier model and the experimental results of [2], that small clusters of cells can chemotax, even if single cells cannot. However, while our minimal model predicts that both velocity and chemotactic index increase as cluster size increases, we find that adaptation to and amplification of the chemoattractant signal can lead to cluster velocity to decrease and chemotactic index to saturate at a value less than one at large cluster sizes. This effect can arise purely from the finite rate of contact mediated diffusive transfer of chemicals between contacting cells. However, even if transfer is fast, if the cell’s response to the signal is switchlike, cluster velocities can be non-monotonic and chemotactic indices can saturate. This non-monotonic behavior is a sign of the way in which the cluster processes the chemoattractant signal. Non-monotonicity of cluster velocity was recently observed in border cell chemotaxis, and was interpreted in terms of additional hydrodynamic resistance at large cluster sizes [8]. Our results here show that alternate mechanisms, like amplification, could potentially also create this qualitative signature, and should be considered.

How do our results compare to experimental data? Theveneau et al. [2] find that chemotactic indices of small (2–3 cell) and large clusters of neural crest cells are similar, but do not observe a large variation in cluster speed. Within our models with adaptation and amplification in adherent clusters, we see that chemotactic indices of 7 cell clusters and 61 cell clusters are often similar—but we generally observe that 2–3 cell clusters have smaller chemotactic indices. This is in part because of orientational averaging—under our assumption that single cells do not chemotax, pairs of cells have a chemotactic behavior that varies strongly with orientation [7]. However, we highlight some difficulties with a direct comparison between model and experiment in this case. First, if cells have a distribution of CIL strengths $\bar{\beta }$, we might expect that cells with higher CIL strength $\bar{\beta }$ form smaller clusters; thus the strength of the collective guidance mechanism could be different between small and large clusters. Secondly, the concentration of the chemoattractant Sdf1 is not well-characterized in the bead assay used by [2]; more extensive studies using microfluidic chambers would aid in pinpointing differences between our model and experiments in neural crest. Non-monotonicity can also make experimental results difficult to interpret. Within our model with adaptation and amplification, a given cluster could have either a larger or smaller velocity than a smaller cluster, depending on the sizes of the clusters and the details of the adaptation mechanism (e.g. the rate of diffusion of the inhibitor). This suggests that solely comparing large and small clusters could potentially be misleading, and in general more detailed experiments as a function of cluster size are needed.

How generic is the result that sufficiently large tightly bound clusters decrease in speed? We have found that this occurs both with adaptation and switch-like amplification, but not with the minimal model of [7], where clusters will break apart if they are not sufficiently tightly bound. We expect this behavior to be relatively broadly present in sufficiently large, and sufficiently tightly bound clusters. In collective guidance in tightly adherent clusters, there is a tug-of-war, and the cluster is under tension, and would tear itself apart in the absence of the strong adhesion. In order for the cluster to maintain its speed as the number of cells increases, which increases friction between the cluster and the surface, the difference in protrusion strength between the front and back must also increase. In adaptation and switchlike amplification, this increase in protrusion strength is slow or absent, and the cluster slows at large *N*. However, if the protrusion strength *β*^*i*^ increases with cluster size, it will eventually overcome cell-cell adhesion—and we expect the cluster to eventually scatter. This argument is not specific to the models we have studied, but should generalize to any model where the cluster is under tension and driven solely by the edge: large tightly adherent clusters will either scatter as the edge force increases, or slow, if the edge can no longer robustly pull the entire cluster.

Our model for strongly adherent clusters has assumed that the primary driver of the dynamics of strongly adherent clusters are cells at the edge. This is consistent with the observations of [2] on neural crest, who observe that only the edge cells develop strong protrusions: there are no cryptic protrusions. In small (2–30 cells) adherent clusters of epithelial cells, traction forces are also mainly found to be large at the edge [70–72]. However, in other systems, most notably the classic example of migration of large epithelial sheets in a wound healing geometry, traction forces are also exerted significantly away from the edge [73]. If these interior traction forces are relevant, we would expect the scaling of cluster velocity with cluster size to be significantly altered. Ultimately, experimental traction force measurements may be crucial in determining whether our assumption of edge-driven dynamics is appropriate; however, this assumption is consistent with the currently available data. We have also recently shown that a similar model of CIL can reproduce some of this traction force data, lending support to our assumptions here [74].

Adaptation in single-cell chemoresponse is a ubiquitous and well-tested principle, but its existence is not established for clusters of neural crest or lymphocytes; applying a step response would be a straightforward test of adaptation, and we would expect protrusions and traction forces to peak and then adapt (Fig 2A). We argue that the gap junction-mediated LEGI model we have used is a reasonable expectation for collective signal processing. Recent papers have independently suggested that gap junctions play a role in gradient sensing and proposed a similar LEGI model [4, 9], though solely in one dimension, and without any effects of CIL, cell motility, or amplification. Our results suggest that gap-junction mediated gradient sensing across the cluster may be effective—though with characteristic effects on the cluster velocity, as discussed above. As noted earlier, our hypothesis of gap junction mediated communication is consistent with the experimental observation that gap junctions modulate neural crest cell motility *in vivo* [35, 36].

In this paper, we also modeled the possibility that the coherence of the cluster is not provided by strong physical adhesion, but rather by chemoattraction to a secreted signal, i.e. co-attraction. This co-attraction mechanism is known to be relevant in neural crest [20] (and see also the model [27]). Our model with co-attraction also shows that, consistent with experiments on neural crest [2], that the collective guidance mechanism proposed here can guide cells even with only transient contacts. However, we also see that if co-attraction is too large, new emergent behaviors can appear, including cluster rotation. Persistent rotation of cell clusters is not observed in neural crest, and only transient rotations appear to occur in lymphocytes undergoing collective chemotaxis [1]. Rotating droplets are, however, observed in bacteria and in the social amoeba Dictyostelium discoideum; vortex formation in bacteria has been speculated to also occur in part via chemotaxis to a secreted molecule [63] though chemotaxis is not necessary and may not be relevant in Dictyostelium [75].

Similar to our model of co-attraction, chemotaxis to a secreted molecule and relayed response to a secreted molecule have been modeled extensively in the literature, with varying degrees of biological specificity and detail [27, 63, 76–82]. We note particularly [27, 81, 83] who also focus on neural crest. Our approach here has been to tend toward minimalism where possible, while still respecting the experimental facts on the neural crest explant experiments of [2]. Many of our assumptions could be generalized, including, e.g. explicitly modeling chemical details of the co-attraction sensing [77], or changing our assumption that cells respond equally strongly to weak and strong gradients of co-attractant. Changing the details of the co-attraction model will change the cluster structure, which will quantitatively change the cluster velocity. Our initial studies in this direction have not yet revealed important qualitative changes.

### Potential extensions and comparisons to other models

Other variants of stochastic particle models have been used to model collective cell migration, ranging from models that use single particles to represent cells [30, 53, 63, 84–86] to those that use more detailed representations of cells with either multiple particles or additional details of cell shape [87–91]. Other techniques, such as the Cellular Potts Model [58, 75, 92] and phase field models [23, 93, 94] have also been developed to study collective cell migration with significantly greater levels of detail on the cell’s shape and its internal biochemistry. Because emergent collective guidance has had only limited quantitative models in the past [1, 7], we have chosen our cell models to be as minimal as possible, in an attempt to focus on the essential aspects of collective guidance. Earlier models have been created to study neural crest chemotaxis in vivo [81, 83, 95]; however, these have explicitly described chemotaxis arising from a “follow-the-leader” mechanism where single leader cells can sense a gradient [96], rather than through the collective mechanism we study here, where individual cells need not sense the gradient level.

We also mention that unlike many of the models discussed above, our model does not include an interaction designed to align a cell’s polarity with its neighbors’ motion [53, 63, 75] or its own velocity or displacement [30, 58, 87, 88], and these mechanisms are not necessary for the effects we describe here. Competition between the collective guidance mechanism and alignment mechanisms may be an interesting area for future study.

Our stochastic interacting particle model is relatively simple, which allows us to in some cases derive analytic results [7]. Many extensions of this approach are possible. Our model could be developed further for more quantitative comparisons by careful measurement of single-cell statistics in or out of a chemoattractant gradient [28, 97]; this could lead to nonlinear or anisotropic terms in Eq 2. Our description of contact inhibition of locomotion has also assumed, for simplicity, that contact with both the front and back of the cell is inhibitory; other possibilities may alter the collective dynamics of the cell cluster [23].

### Summary

Our main findings are: 1) Cluster velocity and chemotactic index may reflect internal signal processing, and provide an experimental window into these processes. 2) We expect sufficiently large clusters undergoing collective guidance to either become increasingly slow or break up. 3) Strong adhesion between cells is not necessary for collective guidance to function if cells chemotax to a secreted molecule. 4) A balance of this co-attraction and graded contact inhibition of locomotion are necessary for efficient chemotaxis. 5) Co-attraction may also induce cluster rotation, and we have explicitly characterized the transition to rotation. 6) The combination of cluster rotation and cluster chemotaxis may induce systematic drifts that depend on cluster rotation.

## Supporting Information

S1 MovieClusters with co-attraction and graded CIL can chemotax effectively even without permanent contacts.We show a representative movie of a chemotaxing cluster loosely bound by co-attraction, with $\bar{\beta }=70$ and *χ* = 15. The color map is the co-attractant field *c*(**r**), the blue arrows are the cell polarity **p**^*i*^, and the cells are drawn as black circles. This video corresponds to Fig 5A.(MP4)

S2 MovieClusters with large co-attraction can develop rotational motion.We show a representative movie of a rotating chemotaxing cluster with strong co-attraction and weaker CIL ($\bar{\beta }=37.2$ and *χ* = 83.3). The color map is the co-attractant field *c*(**r**), the blue arrows are the cell polarity **p**^*i*^, and the cells are drawn as black circles. This video corresponds to Fig 5D.(MP4)

S1 Text Supplemental Information. SI Text provides extended derivations of the behavior of the LEGI model in the fast-diffusion limit, the dynamics of the co-attractant *c*(**r**), the behavior of scattering pairs, and a table of parameters.
(PDF)

S1 Code Supplemental Code. Code and documentation to run our simulations is provided in the file camleySIcode.zip.
(ZIP)
