# Supplementary material for: Collective Signal Processing in Cluster Chemotaxis: Roles of Adaptation, Amplification, and Co-attraction in Collective Guidance
Source: PLoS Comput Biol. 2016 Jul 1;12(7):e1005008. doi: 10.1371/journal.pcbi.1005008 (PMC4930173; doi:10.1371/journal.pcbi.1005008)
Supplement: S1 Text — SI Text provides extended derivations of the behavior of the LEGI model in the fast-diffusion limit, the dynamics of the co-attractant c(r), the behavior of scattering pairs, and a table of parameters. (PDF) [file pcbi.1005008.s003.pdf]

Supplementary Information: Collective signal processing in cluster chemotaxis: roles of adaptation, amplification, and co-attraction in collective guidance

Brian A. Camley Juliane Zimmermann Herbert Levine Wouter-Jan Rappel

1 Proof of perfect adaptation and gradient sensing in limit $k_{-I}/k_D \ll 1$

Our reaction-diffusion model for inhibitor, activator, and response on our network of cells is a direct application of the model of [1] to a network of cells,

$$\partial_t A^i = k_A S(\mathbf{r}^i) - k_{-A} A^i \tag{S1}$$

$$\partial_t I^i = k_I S(\mathbf{r}^i) - k_{-I} I^i - k_D n^i I^i + k_D \sum_{j\sim i} I^j \tag{S2}$$

$$\partial_t R^i = k_R A^i (1 - R^i) - k_{-R} I^i R^i \tag{S3}$$

The steady states of Eq. S1 and Eq. S3 are $A^{i,ss} = \frac{k_A}{k_{-A}} S^i$ and $R^{i,ss} = \frac{A^i/I^i}{A^i/I^i + k_{-R}/k_R}$.

If the signal is constant, the response steady state is independent of the signal: we can see that if $S(\mathbf{r}) = S_0$, $I^{i,ss} = I^{ss}$, and that $I^{i,ss} = \frac{k_I}{k_{-I}} S_0$ and thus $A^{i,ss}/I^{i,ss} = \frac{k_A}{k_{-A}} \frac{k_{-I}}{k_I}$, independent of S_0.

If the signal is *not* uniform, we can find the steady state of Eq. S2 perturbatively in the limit of $\alpha \equiv k_{-I}/k_D \ll 1$. Defining $\iota = k_I/k_{-I}$, we find that the steady state of Eq. S2 obeys

$$\iota \alpha S(\mathbf{r}^i) - \alpha I^{i,ss} + \sum_j \mathcal{L}^{ij} I^{j,ss} = 0 \tag{S4}$$

where $\mathcal{L}^{ij} = C^{ij} - n^i \delta^{ij}$, where C^{ij} is the adjacency matrix of the graph representing cell connections, i.e. $C^{ij} = 1$ if $i \sim j$ and 0 otherwise. $\mathcal{L}^{ij}$ is the "network Laplacian" of the graph [2]. We note that $\sum_j \mathcal{L}^{ij} = \sum_i \mathcal{L}^{ij} = 0$. $\mathcal{L}^{ij}$ is also a W-matrix [3], and by the properties of W-matrices will have a unique (up to normalization) zero eigenvector $\sum_j \mathcal{L}^{ij} V^j = 0$, assuming that the cell cluster is connected. This eigenvector will be constant, $V^j = 1$.

If we write $I^{i,ss} = I^{i,ss}_{(0)} + \alpha I^{i,ss}_{(1)} + \cdots$, by equating powers of α we find that

$$\sum_j \mathcal{L}^{ij} I^{j,ss}_{(0)} = 0 \quad \text{(Zeroth order in } \alpha) \tag{S5}$$

$$\iota S(\mathbf{r}^i) - I^{i,ss}_{(0)} + \sum_j \mathcal{L}^{ij} I^{j,ss}_{(1)} = 0 \quad \text{(First order in } \alpha) \tag{S6}$$

We see from Eq. S5 and the properties of the network Laplacian discussed above that the zeroth order solution $I^{i,ss}_{(0)}$ must be a constant - $I^{i,ss}_{(0)} = \overline{I}$. We can set the overall value of that constant by summing Eq. S6 over i. $\sum_i \mathcal{L}^{ij} = 0$, leading us to conclude

$$\overline{I} = I^{i,ss}_{(0)} = \frac{\iota}{N} \sum_i S(\mathbf{r}^i) = \iota \overline{S} \tag{S7}$$

where $\overline{S}$ is the mean value of S over the cluster. This result, combined with the steady-state for A and the assumption that $R^{i,ss} = \frac{A^i/I^i}{A^i/I^i + k_{-R}/k_R} \approx \frac{k_R}{k_{-R}} \frac{A^i}{I^i}$ yields

$$R^{i,ss} \approx \frac{k_R}{k_{-R}} \frac{k_A}{k_{-A}} \frac{k_{-I}}{k_I} \frac{S(\mathbf{r}^i)}{\overline{S}} \equiv R_0 \frac{S(\mathbf{r}^i)}{\overline{S}} \tag{S8}$$

as quoted in the main paper.

When can this be applied? We expect that in a time t, I will diffuse over $\sim k_D t$ cells; we expect then that if I equilibrates over the cluster within the time scale $1/k_{-I}$, or $k_D/k_{-I} \gg N$, we should have good gradient sensing. This implies that $\alpha \ll N^{-1}$ for observing linear gradient sensing. This is merely the cluster-level version of the conditions applied for the simple one-dimensional gradient sensing model presented in in [1].

2 Details of co-attraction model

We assume that cells secrete a chemical with concentration c, which diffuses in a the extracellular medium with a diffusion coefficient D, and breaks down with a rate k_c. For a single cell at the origin, the equation for $c(\mathbf{r})$ is then:

$$\partial_t c(\mathbf{r},t) = D\nabla^2 c - k_c c + s\delta(\mathbf{r}) \tag{S9}$$

where s is the secretion rate. We assume that the chemical reaches steady state, $\partial_t c = 0$. We can solve this equation via Fourier transformation, finding that (treating our system as two-dimensional)

$$c(\mathbf{r}) = \frac{s}{2\pi D} K_0(r/\ell) \tag{S10}$$

where $\ell^2 = D/k_c$ and $K_0(x)$ is the modified Bessel function of the second kind. By superimposing many solutions, we find that for cells at positions $\mathbf{r}^i$,

$$c(\mathbf{r}^i) = \frac{s}{2\pi D} \sum_j K_0(|\mathbf{r}^i - \mathbf{r}^j|/\ell) \tag{S11}$$

Taking the gradient of this, we find (ignoring the singularity when $i = j$)

$$\nabla c(\mathbf{r}^i) = -\frac{s}{2\pi D\ell} \sum_{j\neq i} K_1(|\mathbf{r}^i - \mathbf{r}^j|/\ell)\hat{\mathbf{r}}^{ij} \tag{S12}$$

We choose $s = 2\pi D\ell$ without loss of generality; this parameter could also be rescaled into the value of χ.

3 Scattering of pairs of cells

We consider two cells that have come into the range of CIL (i.e. separated by a distance D_0). Without loss of generality, we can assume that they are separated along the x axis. In the limit of large $\bar{\beta}$, these collisions will happen quickly (in a time $\sim 1/\bar{\beta}$), so we neglect stochastic noise. We can write the equations of motion of two cells. The rightmost (largest x) cell's position is written as x_r, and the other as x_l:

$$\partial_t x_{r,l} = p_{r,l} \tag{S13}$$

$$\partial_t p_{r,l} = -\tau^{-1} p_{r,l} \pm \beta_{l,r}\Theta(D_0 - |x_r - x_l|) + \chi_{l,r} \tag{S14}$$

We have neglected any physical forces between the cells. This is appropriate if cell-cell adhesion is weak (as in our co-attraction simulations) and cells do not come close enough to have the volume exclusion effect be strong (reasonable if repolarization is fast). The co-attraction creates an effect $\chi_{l,r}$ proportional to χ that moves the cells up the $c(\mathbf{r})$ gradient; in the limit of loosely attached clusters and large $\bar{\beta}$, this can be neglected. We also note that in our minimal model, $\beta_{l,r} = \bar{\beta}S(x_{l,r})$ is dependent on the positions. However, in the large-$\bar{\beta}$ limit, the cells will only move a very small amount before repolarizing and separating – so it is appropriate to approximate $\beta_{l,r}$ as a constant for the time of contact.

We can then write equations for the separation $X = x_r - x_l$ and the difference and the difference and sum of the polarities, $\Delta \equiv p_r - p_l$ and $\Sigma \equiv p_r + p_l$:

$$\partial_t X = \Delta \tag{S15}$$

$$\partial_t \Delta = -\tau^{-1}\Delta + (\beta_r + \beta_l)\,\Theta(D_0 - |X|) \tag{S16}$$

$$\partial_t \Sigma = -\tau^{-1}\Sigma + (\beta_r - \beta_l)\,\Theta(D_0 - |X|) \tag{S17}$$

If we integrate these equations from a collision at $t = 0$ to the point where the cells are separated, t_*, $X(0) = X(t_*) = D_0$, we note that $|X| \leq D_0$ for this entire time and $\Theta(D_0 - |X|) = 1$, so for $t \leq t_*$,

$$X(t) = X(0) + \Delta(0)(1 - e^{-t/\tau}) + (\beta_r + \beta_l)t - (\beta_r + \beta_l)\tau(1 - e^{-t}) \tag{S18}$$

$$\Delta(t) = \Delta e^{-t/\tau} + (\beta_r + \beta_l)\tau(1 - e^{-t/\tau}) \tag{S19}$$

$$\Sigma(t) = \Sigma e^{-t/\tau} + (\beta_r - \beta_l)\tau(1 - e^{-t/\tau}) \tag{S20}$$

We can use the requirement that $X(t_*) = X(0)$ to find t_* using Eq. S18; in the limit where $t_*/\tau \ll 1$, this takes on a particularly simple form:

$$t_* \approx \frac{-2\Delta(0)}{\beta_r + \beta_l}. \tag{S21}$$

We note that $\Delta(0)$ must be negative for t_* to be positive: if the cells are not traveling toward each other, they will merely separate without repolarizing. Using Eq. S19 and Eq. S20, we then find that, assuming that the $\beta_{r,l}$ are large and thus $\tau^{-1}\Sigma(0) \ll (\beta_r - \beta_l)$,

$$\Delta(t_*) \approx -\Delta(0) \tag{S22}$$

$$\Sigma(t_*) \approx \Sigma(0) - 2\Delta(0)\frac{\beta_r - \beta_l}{\beta_r + \beta_l} \tag{S23}$$

We see that in this fast collision limit, if there were no chemical gradient ($\beta_r = \beta_l$), the collisions would be "elastic" and the cells would swap polarities. However, if $\beta_r - \beta_l$ is nonzero, at each collision the cell's mean polarity increases: each collision leads to an effective added velocity toward the chemoattractant.

Crucially, Eq. S23 shows that the increase in the total polarity of the cell pair Σ, which controls the origin of the chemotaxis, depends only on the *relative* change in β across the pair, $\frac{\beta_r - \beta_l}{\beta_r + \beta_l}$. This may provide an explanation for the apparent adaptation. $\beta_{r,l} = \bar{\beta} S(x_{r,l})$ and $\frac{\beta_r - \beta_l}{\beta_r + \beta_l} \approx S_1 D_0/2$, where the approximation holds in an exponential gradient $S(x) = S_1 e^{S_1 x}$ when $S_1 D_0 \ll 1$.

4 Numerical convergence

We earlier selected $\Delta t = 10^{-4}$ in [4] to simulate near-rigid clusters; we found this sufficient in that paper to converge our results to exact analytic results. We have also checked, in the case of amplification, where we do not have good results to compare to, that changing the time step by a factor of two does not change our results beyond the statistical error.

In the co-attraction simulations, the time step was chosen so that results were converged to within statistical error on a test case ($N = 37, \bar{\beta} = 70, \chi = 15$, Fig. S1). We have also checked that changing the time step does not cause important deviations in any of our simulations; even relatively large changes in time step do not create qualitative changes (as long as the numerical integration is stable).

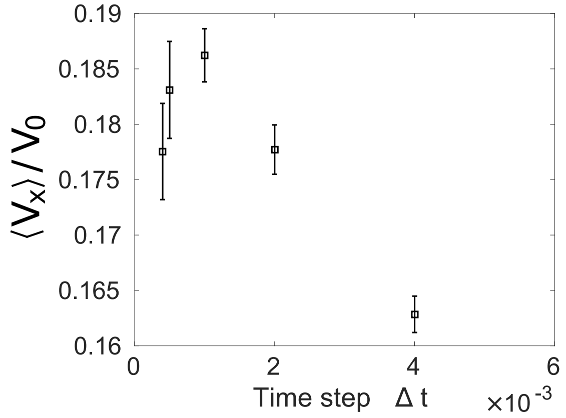

Figure S1: Reducing time steps does not create a statistically significant change beyond $\Delta t = 2 \times 10^{-3}$. $n \geq 100$ trajectories of length 50τ are used for each point. Error bars are one standard deviation of the mean, estimated by a bootstrap method.

5 Table of parameters

Parameter	Name	Value (sim. units)	Justification
τ	Persistence time	1	Estimated from trajectories in [5]
σ	OU noise parameter	1	Required to ensure cell speeds are $\sim$ microns/min as in [5]
$\bar{\beta}$	CIL strength	20 (or as noted)	Used in [4] to ensure cluster speeds are on the order seen in experiments
v_a	Adhesion strength	500 (or as noted)	Chosen in [4] to make clusters perfectly rigid
v_r	Cell repulsion strength	500 (or as noted)	Chosen in [4] to make clusters perfectly rigid
D_0	Maximum interaction length	1.2	Must be roughly the size of a cell as CIL and adhesion are short-range
k_A, k_{-A}	LEGI activator rates	Assumed fast	Minimal assumption
k_R, k_{-R}	LEGI response rates	Assumed fast	Minimal assumption
k_I, k_{-I}	LEGI inhibitor rates	1	Set by constraint that k_I cannot be significantly slower than cell reorientation
k_D	Cell-cell diffusion rate	4	Roughly set by FRAP experiments [6], though dependent on identity of I
λ	Amplification switch threshold	0.01	Chosen so that front, back of clusters are robustly identified (i.e. is smaller than typical difference in R/R_0 across the cluster)
S_0	Signal strength at origin	1 (or as noted)	
ℓ	Degradation length	5	Estimated in [7]
g_0	Gradient threshold value	10^{-5}	Chosen to be small relative to typical gradients $\lvert\nabla c\rvert$ but nonzero
Δt	Time step	10^{-4} (rigid), 10^{-3} (co-att.)	Chosen for convergence (discussed above)

Table S1: **Parameters used**

References

[1] Andre Levchenko and Pablo A Iglesias. Models of eukaryotic gradient sensing: application to chemotaxis of amoebae and neutrophils. *Biophysical Journal*, 82(1):50, 2002.

[2] Hiroya Nakao and Alexander S Mikhailov. Turing patterns in network-organized activator-inhibitor systems. *Nature Physics*, 6(7):544, 2010.

[3] Nicolaas Godfried Van Kampen. *Stochastic Processes in Physics and Chemistry*, volume 1. Elsevier, 1992.

[4] Brian A Camley, Juliane Zimmermann, Herbert Levine, and Wouter-Jan Rappel. Emergent collective chemotaxis without single-cell gradient sensing. *Physical Review Letters*, 116:098101, 2015.

[5] Eric Theveneau, Lorena Marchant, Sei Kuriyama, Mazhar Gull, Barbara Moepps, Maddy Parsons, and Roberto Mayor. Collective chemotaxis requires contact-dependent cell polarity. *Developmental Cell*, 19(1):39, 2010.

[6] Margaret H Wade, James E Trosko, and Melvin Schindler. A fluorescence photobleaching assay of gap junction-mediated communication between human cells. *Science*, 232(4749):525, 1986.

[7] Mae L Woods, Carlos Carmona-Fontaine, Chris P Barnes, Iain D Couzin, Roberto Mayor, and Karen M Page. Directional collective cell migration emerges as a property of cell interactions. *PloS ONE*, 9(9):e104969, 2014.
